# Disease-Modifying Therapies (DMTs) in Pregnant and Lactating Women with Multiple Sclerosis: Analysis of Real-World Data from EudraVigilance Database

**DOI:** 10.3390/ph16111566

**Published:** 2023-11-06

**Authors:** Liberata Sportiello, Raffaella Di Napoli, Nunzia Balzano, Annamaria Mascolo, Rosanna Ruggiero, Luigi Di Costanzo, Davida Monaco, Giorgia Teresa Maniscalco, Annalisa Capuano

**Affiliations:** 1 Campania Regional Centre for Pharmacovigilance and Pharmacoepidemiology, University of Campania “Luigi Vanvitelli”, 80138 Naples, Italy; raffaella.dinapoli@unicampania.it (R.D.N.); nunziabalzano95@gmail.com (N.B.); annamaria.mascolo@unicampania.it (A.M.); rosanna.ruggiero@unicampania.it (R.R.); davida.94@hotmail.it (D.M.); annalisa.capuano@unicampania.it (A.C.); 2 Department of Experimental Medicine, University of Campania “Luigi Vanvitelli”, 80138 Naples, Italy; luisdico86@gmail.com; 3 Multiple Sclerosis Regional Center, “A. Cardarelli” Hospital, 80131 Naples, Italy; gtmaniscalco@libero.it; 4 Neurological Clinic and Stroke Unit, “A. Cardarelli” Hospital, 80131 Naples, Italy

**Keywords:** disease-modifying therapies, pregnancy, breastfeeding, women, adverse drug reaction, safety, database, spontaneous reporting

## Abstract

(1) Background: The purpose of study was to compare the safety profile of glatiramer with natalizumab, alemtuzumab and ocrelizumab in pregnant and lactating women affected by multiple sclerosis (MS). (2) Methods: Individual case safety reports (ICSRs) were retrieved from the European spontaneous reporting system database (EudraVigilance). The reporting odds ratios (RORs) were computed to compare the reporting probability of events between natalizumab, alemtuzumab and ocrelizumab vs. glatiramer. (3) Results: A total of 1236 ICSRs reporting at least one DMT as a suspected drug were selected. More adverse drug reactions (ADRs) unrelated to pregnancy and breastfeeding (*n* = 1171; 32.6%) were reported than ADRs specific to pregnancy and breastfeeding (*n* = 1093; 30.4%). The most frequently reported unrelated ADR was MS relapse. Alemtuzumab and natalizumab seem to have a lower reporting probability of MS relapse compared to glatiramer (ROR 0.17, 95% CI 0.07–0.45 and ROR 0.34, 95% CI 0.20–0.57). Among pregnancy- and breastfeeding-related ADRs, the first most reported event was spontaneous abortion (*n* = 321; 8.9%). Natalizumab and ocrelizumab were associated with a higher reporting probability of spontaneous abortion compared to glatiramer (ROR 2.22, 95% CI 1.58–3.12; ROR 2.18, 95% CI 1.34–3.54, respectively), while alemtuzumab had a lower reporting frequency (ROR 0.32, 95% CI 0.17–0.60). (4) Conclusions: This study did not suggest any strong or new insights for DMTs in this special subpopulation. However, further studies need to be performed.

## 1. Introduction

Multiple sclerosis (MS), an inflammatory demyelinating disease of the central nervous system, is the most common chronic neurological disease with a risk of permanent motor and sensory disability and cognitive deterioration [1]. The course of this disease is unpredictable and changes from person to person [2]. Genetic predisposition, lifestyle and environmental drivers (such as high latitude, female sex, smoking, low vitamin D levels, Epstein–Barr virus infection and obesity) are risk factors for the onset of MS [3]. MS is diagnosed more often in women, with a probability of occurrence about three times higher than that for men. Based on the literature, sex hormones, genes, the immune system and response to immunotherapy may play an important role in this sex difference [4]. In addition, affected people are aged between 20 and 40 years, on average. Therefore, at least 20% to 30% of women with MS are of childbearing age [5]. To date, many studies have shown that MS has no impact on fertility, foetal development or the course of pregnancy and delivery; moreover, pregnancy-related outcomes in MS women are not significantly different from those of the general population [6,7,8]. However, before the end of the 1990s, women with MS were discouraged to undertake pregnancy for reasons related to MS [9]. Thereafter, several studies showed that the activity of MS reduces during pregnancy and significantly increases in the first 3 months post-partum [10,11]. This seems to be due to fluctuations in estrogenic hormone levels in the immune system that could potentially control disease activity [12]. In the last few decades, many disease-modifying therapies (DMTs) have been approved by the European Medicines Agency (EMA) and United States Food and Drug Administration (FDA). However, pre-marketing data did not include relevant information about safety profiles in pregnancy and breastfeeding. Thus, regulatory agencies did not approve any DMTs for pregnant and lactating women. Accordingly, European and American guidelines for MS treatment contraindicated the use of DMTs during pregnancy and breastfeeding [13]. Nevertheless, regulatory agencies continue to steadily review benefit/risk profiles of DMTs. For some older or first-line DMTs (e.g., glatiramer acetate and interferon beta), no relevant safety concerns have emerged and more active MS women sometimes continue to receive them during pregnancy and breastfeeding. In fact, the European regulatory agency removed the contraindication on the label for glatiramer and interferon beta respectively in 2017 and 2019 and allowed their use as clinically needed [12]. These drugs can be referred to as platform therapies in pregnancy and breastfeeding [14]. For some newer or second-line DMTs (e.g., monoclonal antibodies, such as natalizumab (anti-α4 integrin), alemtuzumab (anti-CD20) and ocrelizumab (anti-CD52)) there are limited human data about their safety profiles in pregnancy and breastfeeding. According to the literature, natalizumab seems to have a good benefit/risk balance during conception and the first trimester of pregnancy [15]. However, there are conflicting opinions about maternal and foetal risks. In particular, on the one hand, there is evidence that natalizumab does not appear to increase the risk of spontaneous abortions [16]. On the other hand, natalizumab exposure showed a higher risk of spontaneous abortion or birth defects in pregnant women with MS [17,18]. Moreover, animal data suggested foetal risks requiring a washout period before conception. Therefore, MS women planning pregnancy or with an unplanned confirmed pregnancy were advised to delay starting or discontinue their treatments before conception, during gestation and post-partum, except for cases with high disease activity. Considering that a percentage of pregnancies are unplanned and clinical practice sometimes diverges from official recommendations, and not least that in high-activity MS cases, treatment often needs to be continued based on the clinical choice of the specialist/neurologist, pregnant and lactating women with MS may be exposed to these DMTs [19,20]. Moreover, in the last few years, an increasing proportion of women becoming pregnant during MS therapy has been observed. This different approach is probably due to the decision to bear children after diagnosis, a higher trust in the safety profiles of DMTs gained to date and a greater awareness of the risks related to therapy withdrawal in the case of high disease activity [5]. Regardless, limited evidence is available in the literature on the safety of DMTs in this special female population, which may be due to the low prevalence of MS. Despite several limitations, observational studies and/or pregnancy exposure registries represent relevant tools to investigate this topic thoroughly. Even the pharmacovigilance databases may be a favourable option to provide real-world evidence on the safety data for DMTs in pregnant and lactating women with MS [21,22]. In light of this, herein, we choose to describe and compare the adverse events in pregnant and lactating women with MS receiving one of the platform therapies (glatiramer) or some of the monoclonal antibodies (natalizumab, alemtuzumab and ocrelizumab) using data from the European spontaneous reporting system database, EudraVigilance (EV).

## 2. Results

**Figure 1:**
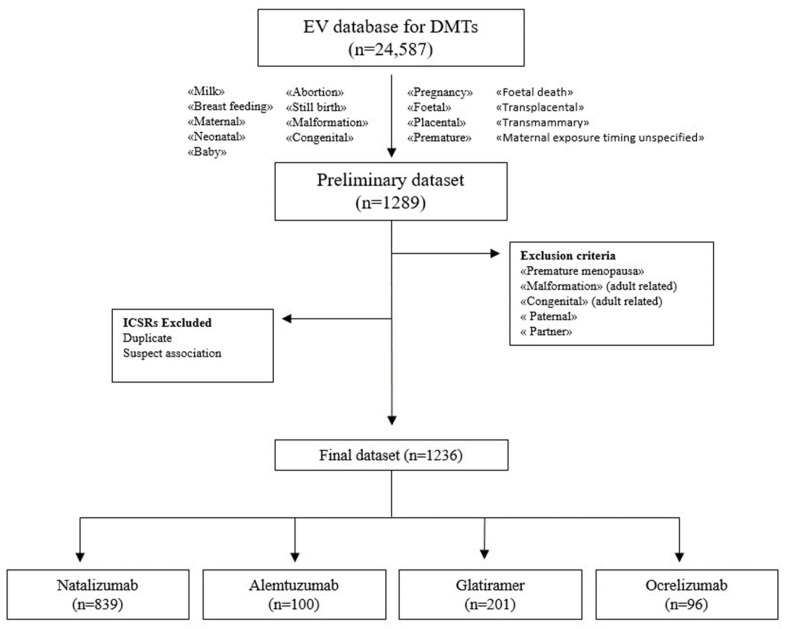
Flowchart of the selection process of ICSRs from the EudraVigilance database.

During the study period, 24,587 individual case safety reports (ICSRs) reporting at least one DMT as a suspected drug were retrieved from the EV: 7582 for natalizumab, 7514 for glatiramer, 5753 for ocrelizumab and 3738 for alemtuzumab. A total of 1236 cases met selection criteria, of which 839 (67.8%) referred to natalizumab, 201 (16.2%) to glatiramer, 100 (8.0%) to alemtuzumab and 96 (7.7%) to ocrelizumab. The selection process of ICSRs from the EV database is shown in the flowchart (Figure 1).

More cases referred to pregnant and lactating women (*n* = 722; 58.4%) than to the offspring (*n* = 188; 15.2%). A high percentage of patients with unknown age was observed (*n* = 326; 26.4%). As expected, almost all ICSRs included pregnant and lactating women aged 18–64 years (58.2%), and only three (0.2%) cases involved adolescent pregnant women (12–17 years). The most reported baby age group was “neonate” for all groups (*n* = 135; 10.9%). Regarding the baby sex, most ICSRs referred to males (*n* = 69; 5.6% vs. *n* = 53; 4.3%), except for ICSRs with alemtuzumab that reported mostly females (*n* = 8; 8.0% vs. *n* = 4; 4.0%). All ICSRs were spontaneous (*n* = 1236, 100.0%), and the primary source was the healthcare professional (*n* = 1051; 85.0%). The most reported geographic origin of ICSRs was the European Economic Area for alemtuzumab (*n* = 67; 67.0%), glatiramer (*n* = 145; 72.1%) and natalizumab (*n* = 529; 63.1%), while the Non-European Economic Area for ocrelizumab (*n* = 55; 57.3%). Of ICSRs, 79.8% (*n* = 986) were serious, while 20.2% (*n* = 250) were not serious. Similar distributions were observed for each DMT. Of ICSRs, 84.8% (*n* = 1048) were codified as “Maternal/Foetal exposure during pregnancy” and/or “Transplacental” (alemtuzumab: *n* = 39; 39.0%; glatiramer: *n* = 180; 89.6%; natalizumab: *n* = 770; 91.8%; ocrelizumab: *n* = 59; 61.5%), while 2.8% (*n* = 34) as “Exposure via breast milk/maternal exposure during breastfeeding” and/or “Transmammary” (alemtuzumab: *n* = 3; 3.0%; glatiramer: *n* = 6; 3.0%; natalizumab: *n* = 22; 2.6%; ocrelizumab: *n* = 3; 3.1%). The overall number of ICSRs related to DMT exposure before pregnancy (preferred terms (PTs) “Maternal exposure before pregnancy/drug exposure before pregnancy” and/or “Transplacental”) was 46 (3.7%), of which 22 (22.0%) by alemtuzumab, 4 (2.0%) by glatiramer and 20 (20.8%) by ocrelizumab. No ICSRs reported pre-gestation exposure with natalizumab. A small percentage of ICSRs reported an unspecified exposure timing (*n* = 13; 1.0%) (PTs “Maternal exposure timing unspecified/foetal exposure timing unspecified”), while the remaining ICSRs (*n* = 95; 7.7%) did not report these PTs. Of ICSRs, 74.6% (*n* = 921) included a DMT without any other suspected drug, while 17.8% reported one suspected drug other than a DMT (*n* = 220). In most ICSRs, concomitant drugs were not reported (*n* = 1013; 82.0%). In the remaining ICSRs, 18.0% reported one/two concomitant drugs. All characteristics of ICSRs for each DMT are shown in Table 1.

### 2.1. Characteristics of Adverse Drug Reactions (ADRs)

All ICSRs (*n* = 1236) were associated with 3590 PTs, of which 1943 were for natalizumab, 720 for glatiramer, 666 for alemtuzumab and 261 for ocrelizumab (Table 2).

The mean number of events per ICSR was 2.9. The “Maternal other ADRs” group was the most frequent (*n* = 1028; 28.6%). The most commonly reported PTs in this group were multiple sclerosis relapse (*n* = 68; 1.9%), haemorrhage (*n* = 15; 0.4%), anaemia (*n* = 13; 0.4%), dyspnoea (*n* = 13; 0.4%) and fatigue (*n* = 13; 0.4%). Focusing on each DMT, we observed mainly pregnancy (*n* = 14; 0.4%), fatigue (*n* = 10; 0.3%), Basedow’s disease (*n* = 10; 0.3%), hyperthyroidism (*n* = 9; 0.2%), lymphocyte count decreased (*n* = 9; 0.2%) and thyroid disorder (*n* = 9; 0.2%) for alemtuzumab; multiple sclerosis relapse (*n* = 30; 0.8%), pregnancy (*n* = 11; 0.3%), injection site erythema (*n* = 9; 0.2%), hypoaesthesia (*n* = 7; 0.2%) and injection site induration (*n* = 7; 0.2%) for glatiramer; multiple sclerosis relapse (*n* = 28; 0.8%), haemorrhage (*n* = 8; 0.2%), anaemia (*n* = 7; 0.2%), urinary tract infection (*n* = 14; 0.4%), hypertension (*n* = 14; 0.4%), and thrombocytopaenia (*n* = 14; 0.4%) for natalizumab; multiple sclerosis relapse (*n* = 5; 0.1%), pregnancy (*n* = 4; 0.1%), haemorrhage (*n* = 3; 0.1%) and vaginal haemorrhage (*n* = 3; 0.1%) for ocrelizumab (Supplementary Table S1). A total of 1093 events (30.4%) were included in the five event groups (Standardised Medical Dictionary for Regulatory Activities (MedDRA) Queries (SMQs)), which are strictly related to pregnancy and breastfeeding both in the mother and in the offspring. Among them, a similar percentage of ICSRs was related to “Termination of pregnancy and risk of abortion” (*n* = 379; 10.6%) and “Pregnancy, labour and delivery complications and risk factors (excluding abortions and stillbirth)” (*n* = 360; 10.0%), with “abortion spontaneous” (*n* = 321; 8.9%) and “Caesarean section” (*n* = 106; 2.9%) as the first PTs, respectively. The “Neonatal disorders” group was reported in 4.5% of cases, characterised mainly by “premature baby” (*n* = 62; 1.7%), “low birth weight baby” (*n* = 25; 0.7%), and “anaemia neonatal” (*n* = 17; 0.5%). In the following two event groups related to “Congenital, familial and genetic disorders” (*n* = 139; 3.9%) and “Foetal disorders” (*n* = 53; 1.5%), the first PT was “trisomy 21” and “foetal growth restriction”, respectively. Although not included in these five specific event groups, 143 events (4.0%) were related to the offspring. Specifically, the most reported PTs were “thrombocytopaenia” (*n* = 9; 0.3%) and “anaemia” (*n* = 4; 0.1%) in the “Neonatal other ADRs” group (*n* = 89; 2.5%); “anaemia” (*n* = 3; 0.1) and “autism spectrum disorder” (*n* = 3; 0.1) in the “Infant other ADRs” group (*n* = 44; 1.2%). Regardless of the event groups, all PTs related to each DMT are reported in the electronic Supplementary Table S1 (Parts I–IX). The group “PTs not indicating ADR” (*n* = 1326; 36.9%) is reported separately in the electronic Supplementary Table S1 (Part X). The overall distribution of non-serious and serious events was 26.5% (*n* = 952) and 73.5% (*n* = 2638), respectively. Considering the reported seriousness criteria, “other medically important condition” (*n* = 1738; 48.4%) and “caused or prolonged the hospitalisation” (*n* = 601; 16.7%) were the most commonly selected. They were followed by “congenital anomaly” (*n* = 203; 5.7%), “results in death” (*n* = 78; 2.2%) and “life threatening” (*n* = 14; 0.4%). Similar distributions were observed for each DMT. However, glatiramer was the only drug reporting “disabling” as serious criteria (*n* = 4; 0.6%). Overall, in half of ADRs (*n* = 1724; 48.0%), the outcome was “unknown” (77.8% for ocrelizumab, 75.3% for glatiramer, 52.4% for alemtuzumab and 32.4% for natalizumab). For the remaining ADRs, the majority reported a positive outcome, such as “recovered/resolved” (*n* = 1326; 36.9%), with the highest percentage for natalizumab (57.5%). The DMT mostly related to a negative outcome was alemtuzumab with “not recovered/not resolved” (34.7%). Seriousness and outcome criteria for each DMT are presented in Table 3.

### 2.2. Reporting Odds Ratio (ROR)

**Figure 2:**
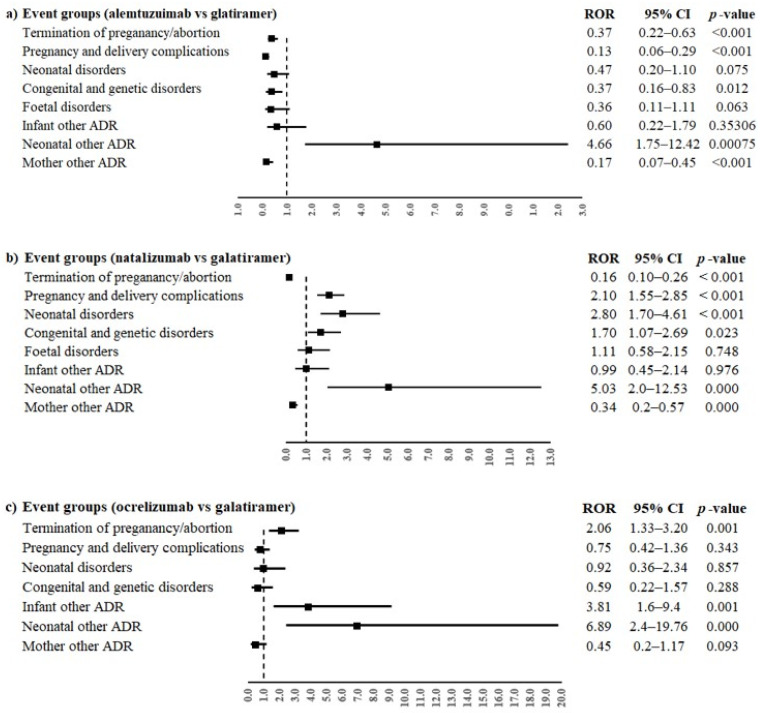
ROR of event groups for the comparisons between monoclonal antibodies ((**a**) alemtuzumab, (**b**) natalizumab and (**c**) ocrelizumab) and glatiramer. ROR, reporting odds ratio; 95% CI, 95% confidence interval.

Alemtuzumab and natalizumab were associated with a lower reporting probability of events belonged to the group “Termination of pregnancy and risk of abortion” compared to glatiramer (ROR 0.37, 95% confidence interval (CI) 0.22–0.63 and ROR 0.16, 95% CI 0.10–0.26, respectively, Figure 2a,b). On the contrary, ocrelizumab was associated with a higher reporting probability than glatiramer for the same event group (ROR 2.06, 95% CI 1.33–3.20, Figure 2c).

**Figure 3:**
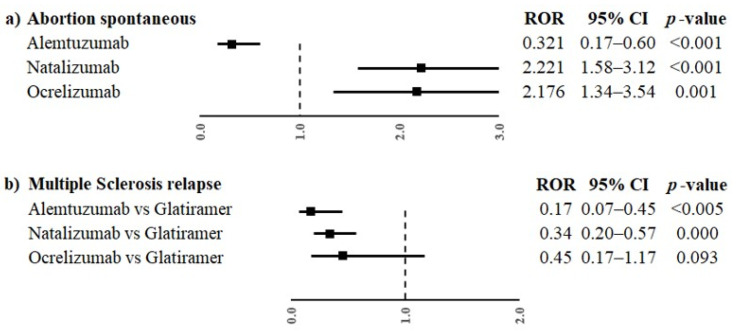
ROR of PTs “abortion spontaneous” (**a**) and “multiple sclerosis relapse” (**b**) for the comparisons between monoclonal antibodies (alemtuzumab, natalizumab and ocrelizumab) and glatiramer. ROR, reporting odds ratio; CI, confidence interval.

Moreover, alemtuzumab was also associated with a lower reporting frequency of events in the groups “Pregnancy, labour and delivery complications and risk factors (excluding abortions and stillbirth)” and “Congenital, familial and genetic disorders” (ROR 0.13, 95% CI 0.06–0.29 and ROR 0.37, 95% CI 0.16–0.83, respectively) compared to glatiramer. On the contrary, natalizumab was associated with a higher reporting probability of events belonging to event groups “Pregnancy, labour and delivery complications and risk factors (excluding abortions and stillbirth)”, “Neonatal disorders” and “Congenital, familial and genetic disorders” compared to glatiramer (ROR 2.10, 95% CI 1.55–3.85; ROR 2.80, 95% CI 1.70–4.61; ROR 1.70, 95% CI 1.07–2-69, respectively). Alemtuzumab, natalizumab and ocrelizumab were associated with a higher reporting probability of events belonging to the group “Neonatal other ADRs” compared to glatiramer (ROR 4.66, 95% CI 1.75–12.42; ROR 5.03, 95% CI 2.0–12.53 and ROR 6.89, 95% CI 2.4–19.76, respectively). Oppositely, alemtuzumab and natalizumab were associated with a lower reporting probability of events belonging to the group “Maternal other ADRs” compared to glatiramer (ROR 0.17, 95% CI 0.07–0.45 and ROR 0.34, 95% CI 0.2–0.57, respectively); ocrelizumab showed a similar result, but without statistical significance. Only ocrelizumab was associated with a higher reporting probability of “Infant other ADRs” than glatiramer (ROR 3.81, 95% CI 1.6–9.4). No other statistically significant difference was observed for the other event groups. The PTs reported in more than three cases for every DMT were “abortion spontaneous” and “multiple sclerosis relapse”. Alemtuzumab had a lower reporting probability of spontaneous abortion compared to glatiramer (ROR 0.32, 95% CI 0.17–0.60), while natalizumab and ocrelizumab had a higher reporting frequency of spontaneous abortion than glatiramer (ROR 2.22, 95% CI 1.58–3.12; ROR 2.18, 95% CI 1.34–3.54, respectively, Figure 3a). Moreover, a statistically significant difference between alemtuzumab or natalizumab and glatiramer was observed for multiple sclerosis relapse (ROR 0.17, 95% CI 0.07–0.45 and ROR 0.34, 95% CI 0.20–0.57), but not for ocrelizumab (ROR 0.45, 95% CI 0.17–1.17, Figure 3b).

## 3. Discussion

In our study, we investigated the maternal and foetal/neonatal adverse events of several DMTs that occurred in multiple sclerosis women during pregnancy and breastfeeding by analysing data from the EV database. Our results suggested that different frequencies of serious but manageable adverse events may occur with DMTs for expectant and lactating women and their babies, requiring appropriate and careful risk monitoring. The choice to evaluate these specific safety concerns was driven by the need to gain more and more scientific evidence on the safety profile of DMTs in pregnant and breastfeeding MS women from the real-life context. A careful evaluation of DMTs’ safety is needed for MS women planning to have children or who are pregnant or are lactating because real-life evidence is meagre [23]. Moreover, the management and treatment of MS in pregnant and lactating women is also complex because many drugs are now available with different routes of administration, mechanisms of action and effectiveness and safety profiles. Furthermore, it is important to evaluate suitably the results of clinical and post-marketing studies in order to appropriately translate data from them to clinical practice. The introduction of DMTs has changed not only the natural history of the disease but also the perspective of pregnancy in women with MS. However, women of childbearing age affected by MS have doubts about the possibility of disease transmission to the child and the impact of drugs, especially DMTs, during pregnancy and breastfeeding [11]. The available literature directing gynaecologists/obstetricians, neurologists and pregnant and lactating patients with MS is increasing and becoming less conflicting. The different safety profiles of DMTs may depend on the target antigen they recognise. Moreover, their ability to cross the placental barrier or to be secreted in milk can also influence their safety profile, representing a potential risk for the developing foetus or neonate or pregnancy complications. For example, alemtuzumab can cross the placental barrier, while natalizumab only minimally crosses it during the first trimester, but its crossing capacity increases during the second and third trimesters [24]. However, in spontaneous reporting, a high number of safety reports for a drug is not necessarily indicative of a worse safety profile because it could also depend on its greater use in clinical practice. For this reason, it is important to apply disproportionality methods. In our study, most ICSRs referred to natalizumab probably because this drug was associated with a higher use during pregnancy than the other DMTs. All DMTs should have the recommended washout periods before planned conception or should be stopped when unplanned pregnancy is confirmed [25,26]. Natalizumab should be stopped 2–3 months before conception. However, natalizumab can be continued in some circumstances (such as in patients with more active or refractory disease) until 30–34 weeks because evidence showed a higher risk of MS relapse during these special conditions and also because it does not cross the placental barrier during the first trimester of pregnancy [25,26,27]. In our study, glatiramer was the second most reported DMT, probably due to its safety in pregnancy and breastfeeding, as evaluated by regulatory agencies. Glatiramer is a complex polypeptide with a large size that does not allow it to cross the placental barrier. There are no data for glatiramer teratogenicity in animal studies [26,28]. In addition, post-marketing clinical studies also confirmed the safety of glatiramer when received in the first trimester [29,30,31,32]. The lower number of ICSRs related to alemtuzumab and ocrelizumab may be because their use in pregnancy is not advised, since they are able to cross the placental barrier [33,34]. The washout periods recommended by the EMA are 4 and 12 months (6 by the FDA) for alemtuzumab and ocrelizumab, respectively [12,35,36]. Moreover, if these DMTs are stopped according to the established recommendations, the potential correlation between those drugs and an adverse event occurring in future is doubtful. This may lead to less reporting. The high percentage (70%) of serious ADRs may be due to several factors, such as the subpopulation of pregnant and lactating women, who are considered vulnerable and consequently associated with more concerns [37]. However, although half of the events (48.0%) did not report the outcome, favourable results predominated over the negative ones (38.7% vs. 13.2%). Moreover, 85.0% of ICSRs were reported by HCPs. Taking into account the seriousness of events, more severe and unexpected clinical occurrences are probably more likely to be reported by HCPs than minor ones, even though they can be coincidental and linked to other causes. Our results showed that most ICSRs (and PTs with a similar trend) were mainly typified by adverse events not related to the special condition of the woman and could also occur in the general population. Among these other maternal adverse events, the sore point was that the first most reported ADR was MS relapse (6.4%). However, the disproportionality analysis showed a reporting probability of MS recurrence that was statistically lower for two of the three newer DMTs analysed (alemtuzumab and natalizumab) than glatiramer. Based on a recent systematic review, a complex and conflicting scenario of the relationship between DMT exposure in relapsing MS women and relapses during and after pregnancy has emerged [38]. However, some evidence suggested the pre-conception exposure of natalizumab was associated with increased risk of relapses during pregnancy [38,39]. In our cases, MS relapses were reported for maternal drug exposure during pregnancy, suggesting the no interruption of natalizumab use during gestation, even if we did not have information about the trimester of pregnancy. In a nationwide observational cohort study of pregnant women with MS exposed to several DMTs and conducted using data from the German MS and Pregnancy Registry (DMSKW), at least one relapse during pregnancy occurred in 19% of women, with a minimum of 6% during the third trimester of pregnancy [40]. The ICSRs related to pregnancy/breastfeeding complications were mostly associated with the “pregnancy” condition both in the mother and the baby compared to the “breastfeeding” condition. This could be due to the possibility for the mother to choose between natural and artificial lactation, based on the need to restart DMT therapy immediately or postpone it to avoid potential damage to the baby [41]. In a cohort of approximately one million pregnant women identified using a wide US healthcare and pharmacy database, MacDonald et al. selected 1649 women with MS of whom 35% were exposed to DMTs during pregnancy. Women with MS with and without DMT use had similar risks (risk ratio) of the pregnancy outcomes (spontaneous abortion, infections, Caesarean section, pre-term delivery, poor foetal growth, pre-eclampsia and major structural malformations) [20]. Based on the literature, a recent study showed that natalizumab exposure resulted in an increased risk of spontaneous abortion (17.3–17.4%), although comparable to that expected in the general Italian population [18]. Moreover, an analysis from the Italian pharmacovigilance database supported this finding; in fact, the authors found a potential safety signal for spontaneous abortion in pregnant women treated with natalizumab (ROR = 208.1; 73.4–590.1) [42]. On the contrary, there is evidence that natalizumab did not induce an increased risk of spontaneous abortion. In fact, a systematic review and meta-analyses of pregnancy and foetal outcomes in women with multiple sclerosis suggested that natalizumab does not appear to increase the risk for spontaneous abortions, pre-term birth or major congenital malformations [16]. Few data are available in literature on alemtuzumab- and ocrelizumab-related spontaneous abortion [35,43]. In our study, among the event groups related to pregnancy, the main one was the termination of pregnancy and risk of abortion, in which the most representative ADR was spontaneous abortion (8.9%). Specifically, natalizumab and ocrelizumab had an about two-fold increased reporting probability of spontaneous abortion compared to glatiramer, while it was lower by 70.0% for alemtuzumab. Because of the spontaneous nature of adverse reaction reporting, the increased reporting probability of spontaneous abortion after natalizumab exposure requires a cautious interpretation. This may reflect the risk to report association between a drug and adverse reaction due to an imbalance in reporting. Generally, spontaneous abortion could be related to several risk factors, and it was difficult to establish the real cause [44]. In several studies based on data of SM National Registries, the overall rate of spontaneous abortion related to DMTs was 6–7%, and it was lower than observed in the general population (8–20%) [39,45]. The may be due to underreporting of abortions, which is often not compulsory in the registries, especially if the pregnancy loss occurred before patient inclusion. A similar reason could also explain our estimate of spontaneous abortion because the phenomenon of underreporting usually also affects the spontaneous reporting system. This means that spontaneous abortion could be underreported by healthcare professionals or patients. In our study, we found a significantly higher probability of reporting other neonatal ADRs related to the three monoclonal antibodies compared to glatiramer. However, the confidence intervals at 95% were very large and this could be explained by the low number of ICSRs retrieved, and it needs to be appropriately interpreted. Natalizumab was mostly associated with thrombocytopenic and anaemic events in neonates but also in infants. In line with our results, Godano et al. showed that natalizumab can cause disorders of haematopoiesis in newborns of patients treated during pregnancy [46]. Also, Proschmann et al. reported a risk of haematological alterations (e.g., pancytopaenia) in neonates because natalizumab can cross the placental barrier before delivery and can be secreted into breast milk [24,47]. Thyroid-related events were mainly reported in ICSRs with alemtuzumab. In the literature, it is known that alemtuzumab can induce a high risk of thyroid disease in the mother (in up to 40% of patients), with Graves’ disease accounting for most of the cases [48], but also in the child (e.g., thyrotoxicosis) [49]. However, unlike other DMTs such as natalizumab, data on pregnancy outcomes in women exposed to alemtuzumab were poor and derived from safety reports of clinical trials [35,50]. The evidence of ocrelizumab safety during pregnancy and breastfeeding was also inadequate, although a risk of B-cell depletion in the foetus or infant was observed after exposure in the second and third trimester [11,24]. In our analysis, we were intrigued by the three cases of autism spectrum disorder (ASD) in the offspring reported after maternal exposure to natalizumab, although no safety alert emerged from a statistical point of view. In the literature, the risk of neurodevelopmental disorders (NDDs) (including specific learning disorders, attention deficit/hyperactivity disorder and ASD) in the progeny is again poorly investigated, but it represents a topic of relevant interest for both clinical and research fields. However, the development of ASD in children can be, at least in part, related to maternal immune activation (MIA)/inflammation during the gestational period. Factors associated with MIA are gestational diabetes mellitus, pre-eclampsia, overweight and obesity, infections, maternal anti-foetal brain antibodies, fever episodes, imbalances in cytokine systems and maternal microbiota [51]. Some studies have been conducted in order to investigate the potential relationship between the childhood NDDs and maternal MS, but no risk emerged [52,53]. Moreover, maternal MS pharmacological treatments in pregnancy did not seem to influence the offspring’s cognitive and behavioural outcomes. However, some controversies should be settled on the negative impact of natalizumab during pregnancy on the neurodevelopment of the foetus, inducing predisposing conditions for childhood NDD diagnosis. Therefore, further animal and human studies are needed to answer this current hot question [45,54].

### Strengths and Limits

EudraVigilance is one of the widest databases in pharmacovigilance, collecting disparate information from different countries and populations. In our study, we could observe DMT-related adverse events in a population subgroup that is typically underrepresented or excluded in pre-authorisation clinical trials and in which the mother is affected by a serious illness (such as multiple sclerosis), which requires treatment, or a condition that untreated may pose significant risk to the foetus [21,55]. Therefore, spontaneous reporting during the post-authorisation phase is one primary source of information on adverse reactions occurring during pregnancy or breastfeeding. However, our study has several limits. First, ICSRs varied in quality and completeness. In fact, our data did not allow us to relate the onset of ADRs with the specific period of exposure in pregnant and lactating women (e.g., first, second and third trimesters of pregnancy) because the information was not specified. These data would have been essential because generally medicinal products may have a different impact at different stages of pregnancy. For example, during the period of organogenesis exposure to a potentially teratogenic agent may induce major malformation, growth retardation or death, while during the second or third trimester the exposure may induce growth retardation, alterations of organ functionality, stillbirth, etc. In our study, we could only identify the exposure as before or during pregnancy. Therefore, the lack of additional useful information (e.g., exact mother’s age, previous abnormal pregnancy outcomes, the drug exposure time, the severity of the underlying MS, a causality assessment, a detailed description of the adverse events or follow-up) made it impossible to infer a causal relationship between a single product and an adverse outcome. Thus, no causality link was performed in this analysis. Although the nature of spontaneous reports from pregnancies infrequently allows the establishment of a causal link, the presence of several reports of a distinct congenital abnormality or any adverse reaction occurring during pregnancy or breastfeeding may constitute a signal and a number of teratogenic agents have been identified in this way [56]. However, spontaneous reporting systems should be improved in order to obtain more detailed data on adverse outcomes related to pregnancy and breastfeeding during drug exposure. Another important limitation is the risk of bias linked to the spontaneous nature of adverse reaction reporting. In particular, we cannot estimate the actually treated quota of patients but only an overview of cases related to adverse drug reactions. Moreover, more than 80% of ICSRs were reported by healthcare professionals; however, we could not distinguish the medical and non-medical reporters in such broad category. We might suppose that the proportion of the medical category was higher than the non-medical one, because this illness is of the expertise of neurologists. However, this is only a hypothesis. Additionally, during the case selection in the EV database, we decided to add several keywords related to pregnancy/breastfeeding outcomes, in addition to the encoding of PTs indicative of pregnancy/breastfeeding, because not all ICSRs could be formally compliant with the regulatory indications suggested in the EMA guideline. However, we may not have selected all cases of our interest. Despite this, a high percentage of ICSRs (92.3%) were appropriately codified as required by the EMA. Another limitation of our study was the exclusion of ICSRs related to paternal SM drug exposure because we wanted merely to focus our attention on pregnant and lactating MS women’s health problems. Therefore, we did not describe all scenarios of DMT exposure. To end, given the limitation of the data source (e.g., the lack of a denominator) and the descriptive nature of our analysis, the interpretation of these results requires caution.

## 4. Materials and Methods

### 4.1. Data Source

EV is the database of the EMA used for reporting ICSRs of suspected ADRs and adverse events following immunisation (AEFIs). The ICSRs are aimed to inform the competent authorities or marketing authorisation holders regarding the adverse events that occur in patients. The access to safety data from EV is open both to those involved in pharmacovigilance activities and the general population (http://www.adrreports.eu/en/index.html, accessed on 6 March 2023). All ICSRs related to a single drug or vaccine can be downloaded in a single Excel file from this database. The information contained in each ICSR included the following: patient age group, sex, type of reporting (spontaneous or not spontaneous), primary source qualification (healthcare professional, non-healthcare professional), geographic origin (European Economic Area, Non-European Economic Area), adverse reaction list, suspect/interacting drug list, concomitant drug list, outcome (recovered/resolved, not recovered/not resolved, recovering/resolving, recovered/resolved with sequelae, fatal, unknown) and seriousness (caused/prolonged hospitalisation, other medically important condition, life threatening, congenital anomaly, disabling, results in death). MedDRA was used in the pharmacovigilance database for coding signs and symptoms of an adverse reaction according to the PTs. MedDRA is a hierarchical system that is organised in five levels, like a matryoshka system, starting with “System Organ Classes” (general level) and ending with the “PTs” (more detailed level). The last level is divided into low-level terms (LLTs) (more specific level). Other MedDRA details are described elsewhere [22,57]. Regarding pregnancy and breastfeeding, the minimum required data for the reports of adverse outcomes (e.g., congenital abnormality, etc.) and data on pregnancy/breastfeeding exposure (with or without ADR) are similar to those required for any ADR report, i.e., an identifiable patient, an identifiable reporter, a suspected ADR and a suspected medicinal product [56]. However, as described in the EMA guideline on good pharmacovigilance practices (GVPs) (Module VI), in the reports of pregnancy- and breastfeeding-related ADRs from spontaneous reporting or other sources, the “route of administration” should be encoded as “transplacental” or “transmammary” and the “reaction/event section” as the MedDRA term “exposure in utero” or “drug exposure via breast milk”, respectively [37].

### 4.2. Data Retrieval

We recovered all ICSRs related to each selected drug for MS (glatiramer, alemtuzumab, natalizumab, ocrelizumab) from the EV website for the period from 1 January 2019 to 31 December 2022. In a unique Excel file, we shared the information of all selected DMTs. Firstly, to identify the ICSRs related to pregnancy and breastfeeding, we carried out an initial selection by searching the PTs “maternal/foetal exposure in/during/before pregnancy”/“foetal exposure timing unspecified” or “exposure during breastfeeding/via breast milk” and then several PTs related to pregnancy and breastfeeding outcomes (including the words “foetal”, “placental”, “premature”, “milk”, “breastfeeding”, “maternal”, “neonatal”, “baby”, “abortion”, “still birth”, “malformation”, “congenital” and “foetal death”), in the column of the PT list. In addition, we searched the terms “transplacental”, “transmammary”, “maternal exposure timing unspecified” in the column related to the suspected drug list (where the information on administration route was reported). In an ICSR, the patient affected by an adverse event due to maternal SM drug exposure during pregnancy or breastfeeding could be the mother, the foetus or the newborn. Secondarily, we excluded the ICSRs with PTs referring to adult age conflicting with our study aim (e.g., including words “premature”, “malformation”, “congenital”) or related to paternal SM drug exposure.

### 4.3. Data Analyses

For each DMT, we categorised all ICSRs for age, baby sex, type of reporting, primary source qualification, primary source country for regulatory purposes and number of suspected or concomitant drugs. When the information in each category was not available, it was indicated as “not specified”. Regarding to ADRs, all PTs were distributed in “event groups”. According to the SMQs, we used the SMQ “Pregnancy and neonatal topics” (divided into five sub-SMQs). Thus, we considered the following groups: (1) “Termination of pregnancy and risk of abortion”; (2) “Pregnancy, labour and delivery complications and risk factors (excluding abortions and stillbirth)”; (3) “Neonatal disorders”; (4) “Congenital, familial and genetic disorders” and (5) “Foetal disorders”. For the PTs not included in these SMQs, we considered other four groups: (6) “Foetal other ADRs”; (7) “Neonatal other ADRs”; (8) “Infant other ADRs” and (9) “Maternal other ADRs”, if they occurred in the foetus, the neonate, the infant or the pregnant or lactating woman, respectively. Lastly, all other PTs not indicative of an ADR were tabled as “PTs not indicating ADRs” separately from the other groups. In this group, we included the MedDRA terms “exposure in utero” or “drug exposure via breast milk” (and similar terms, such as drug exposure before pregnancy, exposure during pregnancy, foetal exposure during pregnancy, foetal exposure timing unspecified, maternal exposure before pregnancy, maternal exposure during pregnancy, maternal exposure timing unspecified, exposure via breast milk, maternal exposure during breast feeding) used to codify these women’s special conditions and those PTs which represent conditions predisposing the onset of an ADR but not the ADR itself. The four most reported PTs for each event group were estimated based on the total number of PTs for all DMTs and then distributed for each DMT. All data analyses were carried out using Excel (Excel, Microsoft 365 office) and the statistical software R Studio (version 4.2.3, R Development Core Team).

### 4.4. Disproportionality Analyses

We conducted a disproportionality analysis applying the ROR and its 95% CI. This method was used to compare the reporting frequency for every event group for each newer DMT (natalizumab, alemtuzumab and ocrelizumab) vs. the older one (glatiramer) considered as the reference drug. Regarding the PTs, we computed ROR and its 95% CI only for those PTs reported at least in 3 cases for each DMT. The ROR was computed as (a/c)/(b/d): “a” is the number of events reported with the DMT of interest, “c” the number of events reported with the comparator, “b” the number of other events reported with the DMT of interest and “d” the number of other events reported with the comparator. For all data, statistical significance was considered with a *p*-value < 0.05.

### 4.5. Compliance with Ethical Standards

Safety data deriving from the spontaneous reporting system are anonymous and follow ethical standards; therefore, no further ethical measure was required.

## 5. Conclusions

The current study provides an overview of spontaneous reports of adverse events in pregnant and breastfeeding MS women receiving DMTs. Thirty percent of adverse events associated with DMTs were strictly related to pregnancy and breastfeeding complications both in the mother and in the offspring. Natalizumab and ocrelizumab were associated with a higher reporting probability of spontaneous abortion compared to glatiramer, while alemtuzumab had a lower reporting frequency. Moreover, glatiramer seemed to have a higher risk of multiple sclerosis relapse compared to alemtuzumab and natalizumab. In agreement with available data in the literature, the results of this post-marketing analysis did not suggest any strong and new insights for DMTs in this special population. Given the above-mentioned limitations of this analysis, further studies need to be performed to better investigate the safety profile of DMTs.

## Figures and Tables

**Table 1:** Characteristics of ICSRs related to maternal exposure during pregnancy and breastfeeding to drugs for multiple sclerosis (MS) reported in EudraVigilance from 1 January 2019 to 31 December 2022.

Event Groups	Glatiramer (*n* = 201)	Alemtuzumab (*n* = 100)	Natalizumab (*n* = 839)	Ocrelizumab (*n* = 96)	Total (*n* = 1236)
Child age group					
Foetus	2 (1.0)	3 (3.0)	5 (0.6)	5 (5.2)	15 (1.2)
Neonate (0–1 m)	13 (6.5)	8 (8.0)	107 (12.8)	7 (7.3)	135 (10.9)
Infant (2 m–2 y)	6 (3.0)	4 (4.0)	25 (3.0)	3 (3.1)	38 (3.1)
Mother age group					
12–17 Years	1 (0.5)	0 (0)	2 (0.2)	0 (0)	3 (0.2)
18–64 Years	126 (62.7)	72 (72.0)	481 (57.3)	40 (41.7)	719 (58.2)
Not specified age group					
Not specified	53 (26.4)	13 (13.0)	219 (26.1)	41 (42.7)	326 (26.4)
Baby sex					
Female	6 (3.0)	8 (8.0)	35 (4.2)	4 (4.2)	53 (4.3)
Male	12 (6.0)	4 (4.0)	45 (5.4)	8 (8.3)	69 (5.6)
Not specified	3 (1.5)	3 (3.0)	57 (6.8)	3 (3.1)	66 (5.3)
Type of reporting					
Spontaneous	201 (100.02)	100 (100.0)	839 (100.0)	96 (100.0)	1236 (100.0)
Not spontaneous	0 (0)	0 (0)	0 (0)	0 (0)	0 (0)
Primary source qualification					
Healthcare professional	117 (58.2)	84 (84.0)	766 (91.3)	84 (87.5)	1051 (85.0)
Non-healthcare professional	84 (41.8)	16 (16.0)	73 (8.7)	12 (12.5)	185 (15.0)
Primary source country					
European economic area	145 (72.1)	67 (67.0)	529 (63.1)	41 (42.7)	782 (63.3)
Non-European economic area	56 (27.9)	33 (33.0)	310 (36.9)	55 (57.3)	454 (36.7)
Seriousness					
Not serious	36 (17.9)	31 (31.0)	168 (20.0)	15 (15.6)	250 (20.2)
Serious	165 (82.1)	69 (69.0)	671 (80.0)	81 (84.4)	986 (79.8)
Type of exposure					
Maternal/Foetal exposure during pregnancy or transplacental route	180 (89.6)	39 (39.0)	770 (91.8)	59 (61.5)	1048 (84.8)
Exposure via breast milk/during breast feeding or transmammary route	6 (3.0)	3 (3.0)	22 (2.6)	3 (3.1)	34 (2.8)
Maternal/Drug exposure before pregnancy	4 (2.0)	22 (22.0)	-	20 (20.8)	46 (3.7)
Maternal/Foetal exposure timing unspecified or transplacental route	-	4 (4.0)	2 (0.2)	7 (7.3)	13 (1.0)
Not specified	11 (5.4)	32 (32.0)	45 (5.4)	7 (7.3)	95 (7.7)
Suspect drug(s) other than DMT					
0	150 (74.6)	85 (85.0)	604 (72.0)	82 (85.4)	921 (74.6)
1	28 (13.9)	6 (6.0)	178 (21.2)	8 (8.3)	220 (17.8)
2	16 (8.0)	4 (4.0)	46 (5.5)	6 (6.3)	72 (5.8)
3	1 (0.5)	3 (3.0)	6 (0.7)	0 (0)	10 (0.8)
≥4	6 (3.0)	2 (2.0)	5 (0.6)	0 (0)	13 (1.1)
Concomitant drug(s)					
1	10 (5.0)	7 (7.0)	49 (5.8)	5 (5.2)	71 (5.7)
2	8 (4.0)	3 (3.0)	36 (4.3)	11 (11.5)	58 (4.7)
3	4 (2.0)	4 (4.0)	21 (2.5)	1 (1.0)	30 (2.4)
4	1 (0.5)	4 (4.0)	12 (1.4)	3 (3.1)	20 (1.6)
≥5	4 (2.0)	10 (10.0)	24 (2.9)	6 (6.3)	44 (3.6)
Not reported	174 (86.6)	72 (72.0)	697 (83.1)	70 (72.9)	1013 (82.0)

Data are expressed as *n* (%).

**Table 2:** Adverse Drug Reactions (ADRs) related to maternal exposure during pregnancy and breastfeeding to drugs for multiple sclerosis (MS) distributed by Event Groups and Preferred Terms.

Event Groups	Glatiramer (*n* = 720)	Alemtuzumab (*n* = 666)	Natalizumab (*n* = 1943)	Ocrelizumab (*n* = 261)	Total (*n* = 3590) § *
Maternal other ADRs	254 (35.3)	501 (75.2)	204 (10.5)	69 (26.4)	1028 (28.6)
Multiple sclerosis relapse	30 (4.2)	5 (0.7)	28 (1.4)	5 (1.9)	68 (1.9)
Haemorrhage	2 (0.3)	2 (0.3)	8 (0.4)	3 (1.1)	15 (0.4)
Anaemia	4 (0.6)	2 (0.3)	7 (0.4)	-	13 (0.4)
Dyspnoea	4 (0.6)	7 (1.1)	1 (0.1)	1 (0.4)	13 (0.4)
Termination of pregnancy and risk of abortion	55 (7.6)	20 (3.0)	266 (13.7)	38 (14.6)	379 (10.6)
Abortion spontaneous	42 (5.8)	13 (2.0)	235 (12.1)	31 (11.9)	321 (8.9)
Abortion	6 (0.8)	2 (0.3)	8 (0.4)	-	16 (0.4)
Abortion missed	3 (0.4)	-	6 (0.3)	3 (1.1)	12 (0.3)
Foetal death	1 (0.1)	1 (0.2)	2 (0.1)	2 (0.8)	6 (0.2)
Pregnancy, labour and delivery complications and risk factors (excl abortions and stillbirth)	54 (7.5)	7 (1.1)	284 (14.6)	15 (5.7)	360 (10.0)
Caesarean section	13 (1.8)	-	93 (4.8)	-	106 (2.9)
Premature delivery	2 (0.3)	-	35 (1.8)	3 (1.1)	40 (1.1)
Gestational diabetes	6 (0.8)	1 (0.2)	8 (0.4)	3 (1.1)	18 (0.5)
Pre-eclampsia	1 (0.1)	-	16 (0.8)	-	17 (0.5)
Neonatal disorders	18 (2.5)	8 (1.2)	130 (6.7)	6 (2.3)	162 (4.5)
Premature baby	5 (0.7)	1 (0.2)	52 (2.7)	4 (1.5)	62 (1.7)
Low birth weight baby	1 (0.1)	-	24 (1.2)	-	25 (0.7)
Anaemia neonatal	-	-	16 (0.8)	1 (0.4)	17 (0.5)
Jaundice neonatal	1 (0.1)	-	5 (0.3)	-	6 (0.2)
Congenital, familial and genetic disorders	23 (3.2)	8 (1.2)	103 (5.3)	5 (1.9)	139 (3.9)
Trisomy 21	-	-	6 (0.3)	1 (0.4)	7 (0.2)
Atrial septal defect	2 (0.3)	1 (0.2)	3 (0.2)	-	6 (0.2)
Ventricular septal defect	1 (0.1)	-	4 (0.2)	-	5 (0.1)
Talipes	-	-	4 (0.2)	-	4 (0.1)
Neonatal other ADRs	3 (0.4)	18 (2.7)	56 (2.9)	12 (4.6)	89 (2.5)
Thrombocytopaenia	-	-	9 (0.5)	-	9 (0.3)
Anaemia	-	-	4 (0.2)	-	4 (0.1)
Platelet count decreased	-	-	3 (0.2)	-	3 (0.1)
Anti-thyroid antibody positive	-	2 (0.3)	-	-	2 (0.06)
Foetal disorders	12 (1.7)	4 (0.6)	36 (1.9)	1 (0.4)	53 (1.5)
Foetal growth restriction	5 (0.7)	3 (0.5)	10 (0.5)	1 (0.4)	19 (0.5)
Foetal heart rate abnormal	2 (0.3)	-	5 (0.3)	-	7 (0.2)
Foetal malformation	2 (0.3)	-	5 (0.3)	-	7 (0.2)
Foetal distress syndrome	-	-	4 (0.2)	-	4 (0.1)
Infant other ADRs	9 (1.3)	5 (0.8)	20 (7.7)	10 (3.8)	44 (1.2)
Anaemia	-	-	3 (0.2)	-	3 (0.1)
Autism spectrum disorder	-	-	3 (0.2)	-	3 (0.1)
COVID-19	2 (0.3)	-	-	1 (0.4)	3 (0.1)
Blood thyroid-stimulating hormone decreased	-	2 (0.3)	-	-	2 (0.1)
Foetal other ADRs	-	6 (0.9)	-	4 (1.5)	10 (0.3)
Bladder dilatation	-	-	-	1 (0.4)	1 (0.0)
Cerebral calcification	-	1 (0.2)	-	-	1 (0.0)
Cerebral cyst	-	1 (0.2)	-	-	1 (0.0)
Cerebral ventricle dilatation	-	1 (0.2)	-	-	1 (0.0)

§ In this Table, the first four most-reported Preferred Terms are listed; All PTs for each event group are reported in the electronic Supplementary Table S1 (Parts I–IX). * The group “PTs not indicating ADRs” is tabled separately in the electronic Supplementary Table S1 (Part X). Data are expressed as *n* (%).

**Table 3:** Seriousness and outcome of ADRs (as PTs) related to maternal exposure during pregnancy and breastfeeding to drugs for multiple sclerosis (MS) reported in EudraVigilance from 1 January 2019 to 31 December 2022.

	Glatiramer (*n* = 720)	Alemtuzumab (*n* = 666)	Natalizumab (*n* = 1943)	Ocrelizumab (*n* = 261)	Total (*n* = 3590)
Seriousness					
Results in death	2 (0.3)	1 (0.2)	67 (3.4)	8 (3.1)	78 (2.2)
Life threatening	1 (0.1)	3 (0.5)	10 (0.5)	0 (0)	14 (0.4)
Caused/prolonged hospitalisation	141 (19.6)	99 (14.9)	334 (17.2)	27 (10.3)	601 (16.7)
Disabling	4 (0.6)	0 (0)	0 (0)	0 (0)	4 (0.1)
Congenital anomaly	22 (3.1)	3 (0.5)	171 (8.8)	7 (2.7)	203 (5.7)
Other medically important condition	341 (47.4)	330 (49.5)	978 (50.3)	89 (34.1)	1738 (48.4)
Not serious	209 (29.0)	230 (34.5)	383 (19.7)	130 (49.8)	952 (26.5)
Outcome					
Recovered/Resolved	108 (15.0)	64 (9.6)	1117 (57.5)	37 (14.2)	1326 (36.9)
Recovering/Resolving	14 (1.9)	21 (3.2)	9 (0.5)	0 (0)	44 (1.2)
Recovered/Resolved with sequelae	9 (1.3)	0 (0)	11 (0.6)	2 (0.8)	22 (0.6)
Not recovered/not resolved	45 (6.3)	231 (34.7)	109 (5.6)	11 (4.2)	396 (11.0)
Fatal	2 (0.3)	1 (0.2)	67 (3.4)	8 (3.1)	78 (2.2)
Unknown	542 (75.3)	349 (52.4)	630 (32.4)	203 (77.8)	1724 (48.0)

Data are expressed as *n* (%).

## Data Availability

European pharmacovigilance data are available at www.adrreports.eu (accessed on 6 March 2023).

## Supplementary Materials

The following supporting information can be downloaded at: https://www.mdpi.com/article/10.3390/ph16111566/s1, Table S1. Preferred Terms reported with alemtuzumab, glatiramer, natalizumab and ocrelizumab in Individual Case Safety Reports related to pregnancy and neonatal topics of the EudraVigilance database.
