# Supplementary material for: Disease-Modifying Therapies (DMTs) in Pregnant and Lactating Women with Multiple Sclerosis: Analysis of Real-World Data from EudraVigilance Database
Source: Pharmaceuticals (Basel). 2023 Nov 6;16(11):1566. doi: 10.3390/ph16111566 (PMC10675378; doi:10.3390/ph16111566)
Supplement: Supplementary file 1 [file pharmaceuticals-16-01566-s001.zip › pharmaceuticals-2634452-supplementary.pdf]

*Supplementary Material*

# Disease-modifying therapies (DMTs) in pregnant and lactating women with multiple sclerosis: analysis of real-world data from EudraVigilance database

**Liberata Sportiello[†,*], Raffaella Di Napoli[†], Nunzia Balzano[†]**

† These authors have contributed equally to this work

* **Correspondence**: Corresponding Author: Liberata Sportiello (liberata.sportiello@unicampania.it)

## 1 Supplementary Tables

**Table S1.** Preferred Terms reported with alemtuzumab, glatiramer, natalizumab, and ocrelizumab in Individual Case Safety Reports related to pregnancy and neonatal topics of the Eudravigilance database.

**Part I – Mother other ADRs**

| Mother other ADR | ALEMTUZUMAB (N=501) | GLATIRAMER (N=254) | NATALIZUMAB (N=204) | OCRELIZUMAB (N=69) | Overall (N=1,028) |
|---|---|---|---|---|---|
| Abdominal discomfort | 1 (0.2%) | 1 (0.4%) | 0 (0%) | 0 (0%) | 2 (0.2%) |
| Abdominal distension | 1 (0.2%) | 1 (0.4%) | 0 (0%) | 0 (0%) | 2 (0.2%) |
| Abdominal pain upper | 2 (0.4%) | 0 (0%) | 0 (0%) | 1 (1.4%) | 3 (0.3%) |
| Acne | 1 (0.2%) | 0 (0%) | 0 (0%) | 0 (0%) | 1 (0.1%) |
| Alanine aminotransferase increased | 1 (0.2%) | 0 (0%) | 0 (0%) | 0 (0%) | 1 (0.1%) |
| Alopecia | 2 (0.4%) | 1 (0.4%) | 0 (0%) | 0 (0%) | 3 (0.3%) |

| | | | | | |
|---|---|---|---|---|---|
| Anaemia | 2 (0.4%) | 4 (1.6%) | 7 (3.4%) | 0 (0%) | 13 (1.3%) |
| Anti-thyroid antibody positive | 6 (1.2%) | 0 (0%) | 0 (0%) | 0 (0%) | 6 (0.6%) |
| Anxiety | 4 (0.8%) | 1 (0.4%) | 0 (0%) | 0 (0%) | 5 (0.5%) |
| Aphasia | 2 (0.4%) | 0 (0%) | 0 (0%) | 0 (0%) | 2 (0.2%) |
| Arthralgia | 2 (0.4%) | 3 (1.2%) | 0 (0%) | 0 (0%) | 5 (0.5%) |
| Asthenia | 3 (0.6%) | 2 (0.8%) | 1 (0.5%) | 0 (0%) | 6 (0.6%) |
| Autoimmune myocarditis | 1 (0.2%) | 0 (0%) | 0 (0%) | 0 (0%) | 1 (0.1%) |
| Autoimmune thyroiditis | 3 (0.6%) | 0 (0%) | 0 (0%) | 0 (0%) | 3 (0.3%) |
| Back pain | 2 (0.4%) | 3 (1.2%) | 1 (0.5%) | 0 (0%) | 6 (0.6%) |
| Bacterial test | 1 (0.2%) | 0 (0%) | 0 (0%) | 0 (0%) | 1 (0.1%) |
| Bacterial test positive | 2 (0.4%) | 0 (0%) | 0 (0%) | 0 (0%) | 2 (0.2%) |
| Band neutrophil count increased | 1 (0.2%) | 0 (0%) | 0 (0%) | 0 (0%) | 1 (0.1%) |
| Band neutrophil percentage increased | 1 (0.2%) | 0 (0%) | 0 (0%) | 0 (0%) | 1 (0.1%) |
| Band sensation | 3 (0.6%) | 0 (0%) | 0 (0%) | 0 (0%) | 3 (0.3%) |
| Basal ganglia haemorrhage | 1 (0.2%) | 0 (0%) | 0 (0%) | 0 (0%) | 1 (0.1%) |
| Basedow's disease | 10 (2.0%) | 0 (0%) | 0 (0%) | 0 (0%) | 10 (1.0%) |
| Blister | 3 (0.6%) | 0 (0%) | 0 (0%) | 0 (0%) | 3 (0.3%) |
| Blood creatinine decreased | 3 (0.6%) | 0 (0%) | 0 (0%) | 0 (0%) | 3 (0.3%) |
| Blood creatinine increased | 3 (0.6%) | 1 (0.4%) | 0 (0%) | 0 (0%) | 4 (0.4%) |
| Blood disorder | 1 (0.2%) | 0 (0%) | 1 (0.5%) | 0 (0%) | 2 (0.2%) |
| Blood iron decreased | 1 (0.2%) | 1 (0.4%) | 0 (0%) | 0 (0%) | 2 (0.2%) |
| Blood ketone body increased | 1 (0.2%) | 0 (0%) | 0 (0%) | 0 (0%) | 1 (0.1%) |
| Blood pressure fluctuation | 1 (0.2%) | 0 (0%) | 0 (0%) | 0 (0%) | 1 (0.1%) |
| Blood pressure increased | 1 (0.2%) | 0 (0%) | 1 (0.5%) | 0 (0%) | 2 (0.2%) |
| Blood sodium decreased | 1 (0.2%) | 0 (0%) | 0 (0%) | 0 (0%) | 1 (0.1%) |
| Blood thyroid stimulating hormone abnormal | 2 (0.4%) | 0 (0%) | 0 (0%) | 0 (0%) | 2 (0.2%) |
| Blood thyroid stimulating hormone decreased | 3 (0.6%) | 0 (0%) | 0 (0%) | 0 (0%) | 3 (0.3%) |
| Blood thyroid stimulating hormone increased | 4 (0.8%) | 0 (0%) | 0 (0%) | 0 (0%) | 4 (0.4%) |
| Blood urine present | 3 (0.6%) | 0 (0%) | 0 (0%) | 0 (0%) | 3 (0.3%) |
| Body temperature increased | 3 (0.6%) | 1 (0.4%) | 0 (0%) | 0 (0%) | 4 (0.4%) |
| Bradycardia | 5 (1.0%) | 0 (0%) | 1 (0.5%) | 0 (0%) | 6 (0.6%) |
| Bronchiolitis | 1 (0.2%) | 0 (0%) | 0 (0%) | 0 (0%) | 1 (0.1%) |

| | | | | | |
|---|---|---|---|---|---|
| Bruxism | 1 (0.2%) | 0 (0%) | 0 (0%) | 0 (0%) | 1 (0.1%) |
| C-reactive protein increased | 3 (0.6%) | 0 (0%) | 1 (0.5%) | 0 (0%) | 4 (0.4%) |
| Cells in urine | 1 (0.2%) | 0 (0%) | 0 (0%) | 0 (0%) | 1 (0.1%) |
| Cerebrovascular accident | 1 (0.2%) | 0 (0%) | 1 (0.5%) | 0 (0%) | 2 (0.2%) |
| Chest discomfort | 1 (0.2%) | 1 (0.4%) | 0 (0%) | 0 (0%) | 2 (0.2%) |
| Chest pain | 5 (1.0%) | 1 (0.4%) | 0 (0%) | 0 (0%) | 6 (0.6%) |
| Chromaturia | 1 (0.2%) | 0 (0%) | 0 (0%) | 0 (0%) | 1 (0.1%) |
| Cognitive disorder | 2 (0.4%) | 0 (0%) | 0 (0%) | 0 (0%) | 2 (0.2%) |
| Constipation | 4 (0.8%) | 1 (0.4%) | 0 (0%) | 1 (1.4%) | 6 (0.6%) |
| Contusion | 1 (0.2%) | 0 (0%) | 0 (0%) | 0 (0%) | 1 (0.1%) |
| Coordination abnormal | 1 (0.2%) | 0 (0%) | 0 (0%) | 0 (0%) | 1 (0.1%) |
| Cough | 3 (0.6%) | 2 (0.8%) | 0 (0%) | 1 (1.4%) | 6 (0.6%) |
| Creatinine urine decreased | 3 (0.6%) | 0 (0%) | 0 (0%) | 0 (0%) | 3 (0.3%) |
| Crystal urine present | 1 (0.2%) | 0 (0%) | 0 (0%) | 0 (0%) | 1 (0.1%) |
| Culture urine positive | 5 (1.0%) | 0 (0%) | 0 (0%) | 0 (0%) | 5 (0.5%) |
| Cytomegalovirus infection | 1 (0.2%) | 0 (0%) | 1 (0.5%) | 0 (0%) | 2 (0.2%) |
| Death | 1 (0.2%) | 0 (0%) | 0 (0%) | 0 (0%) | 1 (0.1%) |
| Decreased appetite | 1 (0.2%) | 1 (0.4%) | 0 (0%) | 0 (0%) | 2 (0.2%) |
| Depression | 3 (0.6%) | 1 (0.4%) | 1 (0.5%) | 1 (1.4%) | 6 (0.6%) |
| Diarrhoea | 6 (1.2%) | 1 (0.4%) | 0 (0%) | 1 (1.4%) | 8 (0.8%) |
| Disorientation | 2 (0.4%) | 0 (0%) | 0 (0%) | 0 (0%) | 2 (0.2%) |
| Dizziness | 5 (1.0%) | 0 (0%) | 0 (0%) | 0 (0%) | 5 (0.5%) |
| Drug ineffective | 3 (0.6%) | 0 (0%) | 1 (0.5%) | 0 (0%) | 4 (0.4%) |
| Dry skin | 2 (0.4%) | 0 (0%) | 0 (0%) | 0 (0%) | 2 (0.2%) |
| Dysarthria | 1 (0.2%) | 0 (0%) | 1 (0.5%) | 0 (0%) | 2 (0.2%) |
| Dysgeusia | 1 (0.2%) | 0 (0%) | 0 (0%) | 0 (0%) | 1 (0.1%) |
| Dyspepsia | 1 (0.2%) | 1 (0.4%) | 0 (0%) | 0 (0%) | 2 (0.2%) |
| Dysphonia | 1 (0.2%) | 0 (0%) | 0 (0%) | 0 (0%) | 1 (0.1%) |
| Dyspnoea | 7 (1.4%) | 4 (1.6%) | 1 (0.5%) | 1 (1.4%) | 13 (1.3%) |
| Electric shock sensation | 1 (0.2%) | 0 (0%) | 0 (0%) | 0 (0%) | 1 (0.1%) |
| Encephalitis autoimmune | 1 (0.2%) | 0 (0%) | 0 (0%) | 0 (0%) | 1 (0.1%) |
| Enterovirus infection | 1 (0.2%) | 0 (0%) | 0 (0%) | 0 (0%) | 1 (0.1%) |

| | | | | | |
|---|---|---|---|---|---|
| Eosinophil count increased | 1 (0.2%) | 0 (0%) | 0 (0%) | 0 (0%) | 1 (0.1%) |
| Epistaxis | 4 (0.8%) | 0 (0%) | 0 (0%) | 0 (0%) | 4 (0.4%) |
| Erythema | 1 (0.2%) | 1 (0.4%) | 0 (0%) | 1 (1.4%) | 3 (0.3%) |
| Erythema migrans | 1 (0.2%) | 0 (0%) | 0 (0%) | 0 (0%) | 1 (0.1%) |
| Exophthalmos | 1 (0.2%) | 0 (0%) | 0 (0%) | 0 (0%) | 1 (0.1%) |
| Extrasystoles | 1 (0.2%) | 0 (0%) | 0 (0%) | 0 (0%) | 1 (0.1%) |
| Eye contusion | 1 (0.2%) | 0 (0%) | 0 (0%) | 0 (0%) | 1 (0.1%) |
| Eye disorder | 1 (0.2%) | 0 (0%) | 1 (0.5%) | 0 (0%) | 2 (0.2%) |
| Fatigue | 10 (2.0%) | 2 (0.8%) | 1 (0.5%) | 0 (0%) | 13 (1.3%) |
| Feeding disorder | 1 (0.2%) | 0 (0%) | 0 (0%) | 0 (0%) | 1 (0.1%) |
| Feeling abnormal | 3 (0.6%) | 2 (0.8%) | 0 (0%) | 0 (0%) | 5 (0.5%) |
| Feeling hot | 3 (0.6%) | 0 (0%) | 0 (0%) | 0 (0%) | 3 (0.3%) |
| Flatulence | 1 (0.2%) | 1 (0.4%) | 0 (0%) | 0 (0%) | 2 (0.2%) |
| Flushing | 1 (0.2%) | 0 (0%) | 0 (0%) | 0 (0%) | 1 (0.1%) |
| Gait disturbance | 1 (0.2%) | 1 (0.4%) | 1 (0.5%) | 0 (0%) | 3 (0.3%) |
| Gastrointestinal sounds abnormal | 1 (0.2%) | 0 (0%) | 0 (0%) | 0 (0%) | 1 (0.1%) |
| Gastrooesophageal reflux disease | 4 (0.8%) | 0 (0%) | 1 (0.5%) | 0 (0%) | 5 (0.5%) |
| Generalised tonic-clonic seizure | 1 (0.2%) | 0 (0%) | 0 (0%) | 0 (0%) | 1 (0.1%) |
| Gingival bleeding | 1 (0.2%) | 0 (0%) | 0 (0%) | 0 (0%) | 1 (0.1%) |
| Goitre | 1 (0.2%) | 0 (0%) | 0 (0%) | 0 (0%) | 1 (0.1%) |
| Haematocrit decreased | 3 (0.6%) | 0 (0%) | 2 (1.0%) | 0 (0%) | 5 (0.5%) |
| Haematoma | 1 (0.2%) | 0 (0%) | 0 (0%) | 0 (0%) | 1 (0.1%) |
| Haemoglobin decreased | 3 (0.6%) | 0 (0%) | 3 (1.5%) | 0 (0%) | 6 (0.6%) |
| Haemorrhage | 2 (0.4%) | 2 (0.8%) | 8 (3.9%) | 3 (4.3%) | 15 (1.5%) |
| Hair texture abnormal | 1 (0.2%) | 0 (0%) | 0 (0%) | 0 (0%) | 1 (0.1%) |
| Headache | 6 (1.2%) | 3 (1.2%) | 0 (0%) | 1 (1.4%) | 10 (1.0%) |
| Heart rate increased | 5 (1.0%) | 0 (0%) | 0 (0%) | 0 (0%) | 5 (0.5%) |
| Heavy menstrual bleeding | 1 (0.2%) | 0 (0%) | 0 (0%) | 0 (0%) | 1 (0.1%) |
| Hemiparaesthesia | 1 (0.2%) | 0 (0%) | 0 (0%) | 0 (0%) | 1 (0.1%) |
| Hemiparesis | 1 (0.2%) | 0 (0%) | 0 (0%) | 0 (0%) | 1 (0.1%) |
| Herpes zoster | 1 (0.2%) | 0 (0%) | 0 (0%) | 1 (1.4%) | 2 (0.2%) |
| Hot flush | 4 (0.8%) | 0 (0%) | 0 (0%) | 0 (0%) | 4 (0.4%) |

| | | | | | |
|---|---|---|---|---|---|
| Hyperhidrosis | 4 (0.8%) | 0 (0%) | 0 (0%) | 0 (0%) | 4 (0.4%) |
| Hypertension | 1 (0.2%) | 0 (0%) | 4 (2.0%) | 0 (0%) | 5 (0.5%) |
| Hyperthyroidism | 9 (1.8%) | 0 (0%) | 0 (0%) | 0 (0%) | 9 (0.9%) |
| Hypertonia | 1 (0.2%) | 0 (0%) | 0 (0%) | 0 (0%) | 1 (0.1%) |
| Hypoaesthesia | 3 (0.6%) | 7 (2.8%) | 0 (0%) | 0 (0%) | 10 (1.0%) |
| Hypohidrosis | 1 (0.2%) | 0 (0%) | 0 (0%) | 0 (0%) | 1 (0.1%) |
| Hypothyroidism | 5 (1.0%) | 0 (0%) | 0 (0%) | 0 (0%) | 5 (0.5%) |
| Immune reconstitution inflammatory syndrome | 1 (0.2%) | 0 (0%) | 0 (0%) | 0 (0%) | 1 (0.1%) |
| Immune thrombocytopenia | 5 (1.0%) | 0 (0%) | 0 (0%) | 0 (0%) | 5 (0.5%) |
| Increased appetite | 3 (0.6%) | 0 (0%) | 0 (0%) | 0 (0%) | 3 (0.3%) |
| Infection | 1 (0.2%) | 1 (0.4%) | 2 (1.0%) | 0 (0%) | 4 (0.4%) |
| Influenza | 1 (0.2%) | 0 (0%) | 2 (1.0%) | 0 (0%) | 3 (0.3%) |
| Infusion site bruising | 1 (0.2%) | 0 (0%) | 0 (0%) | 0 (0%) | 1 (0.1%) |
| Infusion site induration | 1 (0.2%) | 0 (0%) | 0 (0%) | 0 (0%) | 1 (0.1%) |
| Initial insomnia | 1 (0.2%) | 0 (0%) | 0 (0%) | 0 (0%) | 1 (0.1%) |
| Insomnia | 4 (0.8%) | 0 (0%) | 0 (0%) | 0 (0%) | 4 (0.4%) |
| Irritability | 1 (0.2%) | 0 (0%) | 0 (0%) | 0 (0%) | 1 (0.1%) |
| Klebsiella test positive | 1 (0.2%) | 0 (0%) | 0 (0%) | 0 (0%) | 1 (0.1%) |
| Lethargy | 1 (0.2%) | 0 (0%) | 0 (0%) | 0 (0%) | 1 (0.1%) |
| Leukaemia | 1 (0.2%) | 0 (0%) | 0 (0%) | 0 (0%) | 1 (0.1%) |
| Loss of consciousness | 2 (0.4%) | 0 (0%) | 0 (0%) | 0 (0%) | 2 (0.2%) |
| Lower respiratory tract infection | 1 (0.2%) | 0 (0%) | 0 (0%) | 0 (0%) | 1 (0.1%) |
| Lymphadenectomy | 1 (0.2%) | 0 (0%) | 0 (0%) | 0 (0%) | 1 (0.1%) |
| Lymphadenitis | 1 (0.2%) | 0 (0%) | 0 (0%) | 0 (0%) | 1 (0.1%) |
| Lymphocyte count decreased | 9 (1.8%) | 1 (0.4%) | 0 (0%) | 1 (1.4%) | 11 (1.1%) |
| Lymphocyte percentage decreased | 2 (0.4%) | 0 (0%) | 0 (0%) | 0 (0%) | 2 (0.2%) |
| Lymphoid tissue hyperplasia | 1 (0.2%) | 0 (0%) | 0 (0%) | 0 (0%) | 1 (0.1%) |
| Lymphopenia | 1 (0.2%) | 0 (0%) | 0 (0%) | 0 (0%) | 1 (0.1%) |
| Malaise | 4 (0.8%) | 2 (0.8%) | 0 (0%) | 0 (0%) | 6 (0.6%) |
| Mean cell haemoglobin concentration increased | 1 (0.2%) | 0 (0%) | 0 (0%) | 0 (0%) | 1 (0.1%) |

| | | | | | |
|---|---|---|---|---|---|
| Mean cell haemoglobin increased | 1 (0.2%) | 0 (0%) | 0 (0%) | 0 (0%) | 1 (0.1%) |
| Mean cell volume increased | 1 (0.2%) | 0 (0%) | 1 (0.5%) | 0 (0%) | 2 (0.2%) |
| Memory impairment | 1 (0.2%) | 1 (0.4%) | 1 (0.5%) | 0 (0%) | 3 (0.3%) |
| Menstrual disorder | 1 (0.2%) | 0 (0%) | 0 (0%) | 0 (0%) | 1 (0.1%) |
| Menstruation delayed | 1 (0.2%) | 0 (0%) | 0 (0%) | 0 (0%) | 1 (0.1%) |
| Menstruation irregular | 1 (0.2%) | 0 (0%) | 0 (0%) | 0 (0%) | 1 (0.1%) |
| Monocyte count decreased | 1 (0.2%) | 0 (0%) | 0 (0%) | 0 (0%) | 1 (0.1%) |
| Monocyte count increased | 3 (0.6%) | 0 (0%) | 0 (0%) | 0 (0%) | 3 (0.3%) |
| Monocyte percentage increased | 1 (0.2%) | 0 (0%) | 0 (0%) | 0 (0%) | 1 (0.1%) |
| Mood altered | 1 (0.2%) | 0 (0%) | 0 (0%) | 0 (0%) | 1 (0.1%) |
| Motor dysfunction | 1 (0.2%) | 1 (0.4%) | 0 (0%) | 0 (0%) | 2 (0.2%) |
| Multiple sclerosis relapse | 5 (1.0%) | 30 (11.8%) | 28 (13.7%) | 5 (7.2%) | 68 (6.6%) |
| Muscle spasms | 1 (0.2%) | 0 (0%) | 1 (0.5%) | 0 (0%) | 2 (0.2%) |
| Muscle tightness | 1 (0.2%) | 0 (0%) | 0 (0%) | 0 (0%) | 1 (0.1%) |
| Muscular weakness | 3 (0.6%) | 1 (0.4%) | 0 (0%) | 0 (0%) | 4 (0.4%) |
| Musculoskeletal disorder | 1 (0.2%) | 0 (0%) | 0 (0%) | 0 (0%) | 1 (0.1%) |
| Musculoskeletal stiffness | 2 (0.4%) | 2 (0.8%) | 0 (0%) | 0 (0%) | 4 (0.4%) |
| Myalgia | 5 (1.0%) | 0 (0%) | 0 (0%) | 0 (0%) | 5 (0.5%) |
| Myasthenia gravis | 2 (0.4%) | 0 (0%) | 0 (0%) | 0 (0%) | 2 (0.2%) |
| Myxoedema | 1 (0.2%) | 0 (0%) | 0 (0%) | 0 (0%) | 1 (0.1%) |
| Nasal congestion | 3 (0.6%) | 0 (0%) | 0 (0%) | 0 (0%) | 3 (0.3%) |
| Nasal injury | 1 (0.2%) | 0 (0%) | 0 (0%) | 0 (0%) | 1 (0.1%) |
| Nasopharyngitis | 1 (0.2%) | 1 (0.4%) | 0 (0%) | 0 (0%) | 2 (0.2%) |
| Nausea | 8 (1.6%) | 0 (0%) | 2 (1.0%) | 0 (0%) | 10 (1.0%) |
| Nervous system disorder | 1 (0.2%) | 0 (0%) | 0 (0%) | 0 (0%) | 1 (0.1%) |
| Neurodermatitis | 1 (0.2%) | 1 (0.4%) | 0 (0%) | 0 (0%) | 2 (0.2%) |
| Neutropenia | 1 (0.2%) | 0 (0%) | 0 (0%) | 1 (1.4%) | 2 (0.2%) |
| Neutrophil count decreased | 1 (0.2%) | 0 (0%) | 1 (0.5%) | 0 (0%) | 2 (0.2%) |
| Neutrophil count increased | 4 (0.8%) | 0 (0%) | 0 (0%) | 0 (0%) | 4 (0.4%) |
| Neutrophil percentage increased | 1 (0.2%) | 0 (0%) | 0 (0%) | 0 (0%) | 1 (0.1%) |
| Nitrite urine present | 2 (0.4%) | 0 (0%) | 0 (0%) | 0 (0%) | 2 (0.2%) |
| Nocturia | 1 (0.2%) | 0 (0%) | 0 (0%) | 0 (0%) | 1 (0.1%) |

| | | | | | |
|---|---|---|---|---|---|
| Oedema peripheral | 1 (0.2%) | 0 (0%) | 0 (0%) | 0 (0%) | 1 (0.1%) |
| Optic atrophy | 1 (0.2%) | 0 (0%) | 0 (0%) | 0 (0%) | 1 (0.1%) |
| Oral candidiasis | 1 (0.2%) | 0 (0%) | 0 (0%) | 1 (1.4%) | 2 (0.2%) |
| Oropharyngeal pain | 4 (0.8%) | 0 (0%) | 0 (0%) | 0 (0%) | 4 (0.4%) |
| Pain | 2 (0.4%) | 1 (0.4%) | 1 (0.5%) | 1 (1.4%) | 5 (0.5%) |
| Pain in jaw | 3 (0.6%) | 0 (0%) | 0 (0%) | 0 (0%) | 3 (0.3%) |
| Panic attack | 3 (0.6%) | 0 (0%) | 0 (0%) | 0 (0%) | 3 (0.3%) |
| Peripheral swelling | 1 (0.2%) | 1 (0.4%) | 0 (0%) | 1 (1.4%) | 3 (0.3%) |
| Petechiae | 1 (0.2%) | 0 (0%) | 0 (0%) | 0 (0%) | 1 (0.1%) |
| pH urine increased | 1 (0.2%) | 0 (0%) | 0 (0%) | 0 (0%) | 1 (0.1%) |
| Pharyngitis | 1 (0.2%) | 0 (0%) | 0 (0%) | 0 (0%) | 1 (0.1%) |
| Platelet count decreased | 3 (0.6%) | 0 (0%) | 1 (0.5%) | 0 (0%) | 4 (0.4%) |
| Pollakiuria | 2 (0.4%) | 0 (0%) | 0 (0%) | 0 (0%) | 2 (0.2%) |
| Polyarteritis nodosa | 1 (0.2%) | 0 (0%) | 0 (0%) | 0 (0%) | 1 (0.1%) |
| Procalcitonin increased | 1 (0.2%) | 0 (0%) | 0 (0%) | 0 (0%) | 1 (0.1%) |
| Protein total increased | 1 (0.2%) | 0 (0%) | 0 (0%) | 0 (0%) | 1 (0.1%) |
| Pruritus | 2 (0.4%) | 2 (0.8%) | 0 (0%) | 1 (1.4%) | 5 (0.5%) |
| Pulmonary hypertension | 1 (0.2%) | 0 (0%) | 0 (0%) | 0 (0%) | 1 (0.1%) |
| Pyrexia | 4 (0.8%) | 1 (0.4%) | 2 (1.0%) | 0 (0%) | 7 (0.7%) |
| Rash | 4 (0.8%) | 0 (0%) | 1 (0.5%) | 2 (2.9%) | 7 (0.7%) |
| Red blood cell count decreased | 3 (0.6%) | 0 (0%) | 2 (1.0%) | 0 (0%) | 5 (0.5%) |
| Red blood cell count increased | 1 (0.2%) | 0 (0%) | 0 (0%) | 0 (0%) | 1 (0.1%) |
| Red blood cells urine positive | 3 (0.6%) | 0 (0%) | 0 (0%) | 0 (0%) | 3 (0.3%) |
| Red cell distribution width decreased | 2 (0.4%) | 0 (0%) | 0 (0%) | 0 (0%) | 2 (0.2%) |
| Renal pain | 2 (0.4%) | 0 (0%) | 0 (0%) | 0 (0%) | 2 (0.2%) |
| Rhinitis | 2 (0.4%) | 0 (0%) | 0 (0%) | 0 (0%) | 2 (0.2%) |
| Rosacea | 1 (0.2%) | 0 (0%) | 0 (0%) | 0 (0%) | 1 (0.1%) |
| Salivary gland cancer | 1 (0.2%) | 0 (0%) | 0 (0%) | 0 (0%) | 1 (0.1%) |
| Sensation of foreign body | 3 (0.6%) | 0 (0%) | 0 (0%) | 0 (0%) | 3 (0.3%) |
| Sensorimotor disorder | 1 (0.2%) | 0 (0%) | 0 (0%) | 0 (0%) | 1 (0.1%) |
| Sensory disturbance | 1 (0.2%) | 0 (0%) | 0 (0%) | 1 (1.4%) | 2 (0.2%) |
| Sinusitis | 1 (0.2%) | 0 (0%) | 0 (0%) | 0 (0%) | 1 (0.1%) |

| | | | | | |
|---|---|---|---|---|---|
| Skin burning sensation | 3 (0.6%) | 0 (0%) | 0 (0%) | 0 (0%) | 3 (0.3%) |
| Skin discolouration | 1 (0.2%) | 0 (0%) | 0 (0%) | 0 (0%) | 1 (0.1%) |
| Skin disorder | 1 (0.2%) | 0 (0%) | 0 (0%) | 0 (0%) | 1 (0.1%) |
| Skin haemorrhage | 1 (0.2%) | 0 (0%) | 0 (0%) | 0 (0%) | 1 (0.1%) |
| Specific gravity urine decreased | 1 (0.2%) | 0 (0%) | 0 (0%) | 0 (0%) | 1 (0.1%) |
| Speech disorder | 1 (0.2%) | 0 (0%) | 0 (0%) | 0 (0%) | 1 (0.1%) |
| Staphylococcal infection | 1 (0.2%) | 0 (0%) | 0 (0%) | 0 (0%) | 1 (0.1%) |
| Status epilepticus | 1 (0.2%) | 0 (0%) | 0 (0%) | 0 (0%) | 1 (0.1%) |
| Stress cardiomyopathy | 1 (0.2%) | 0 (0%) | 0 (0%) | 0 (0%) | 1 (0.1%) |
| Swelling face | 1 (0.2%) | 1 (0.4%) | 0 (0%) | 0 (0%) | 2 (0.2%) |
| T-cell type acute leukaemia | 1 (0.2%) | 0 (0%) | 0 (0%) | 0 (0%) | 1 (0.1%) |
| Tachycardia | 1 (0.2%) | 0 (0%) | 2 (1.0%) | 0 (0%) | 3 (0.3%) |
| Temperature intolerance | 2 (0.4%) | 0 (0%) | 0 (0%) | 0 (0%) | 2 (0.2%) |
| Thrombocytopenia | 1 (0.2%) | 0 (0%) | 4 (2.0%) | 0 (0%) | 5 (0.5%) |
| Thyroid cyst | 3 (0.6%) | 0 (0%) | 0 (0%) | 0 (0%) | 3 (0.3%) |
| Thyroid disorder | 9 (1.8%) | 0 (0%) | 0 (0%) | 0 (0%) | 9 (0.9%) |
| Thyroid function test abnormal | 4 (0.8%) | 0 (0%) | 0 (0%) | 0 (0%) | 4 (0.4%) |
| Thyroid stimulating immunoglobulin | 1 (0.2%) | 0 (0%) | 0 (0%) | 0 (0%) | 1 (0.1%) |
| Thyroidectomy | 1 (0.2%) | 0 (0%) | 0 (0%) | 0 (0%) | 1 (0.1%) |
| Thyroxine decreased | 1 (0.2%) | 0 (0%) | 0 (0%) | 0 (0%) | 1 (0.1%) |
| Thyroxine free abnormal | 1 (0.2%) | 0 (0%) | 0 (0%) | 0 (0%) | 1 (0.1%) |
| Thyroxine free decreased | 2 (0.4%) | 0 (0%) | 0 (0%) | 0 (0%) | 2 (0.2%) |
| Thyroxine increased | 2 (0.4%) | 0 (0%) | 0 (0%) | 0 (0%) | 2 (0.2%) |
| Tongue discolouration | 1 (0.2%) | 0 (0%) | 0 (0%) | 0 (0%) | 1 (0.1%) |
| Transformation to acute myeloid leukaemia | 1 (0.2%) | 0 (0%) | 0 (0%) | 0 (0%) | 1 (0.1%) |
| Tremor | 5 (1.0%) | 3 (1.2%) | 0 (0%) | 0 (0%) | 8 (0.8%) |
| Tri-iodothyronine free abnormal | 1 (0.2%) | 0 (0%) | 0 (0%) | 0 (0%) | 1 (0.1%) |
| Tri-iodothyronine increased | 1 (0.2%) | 0 (0%) | 0 (0%) | 0 (0%) | 1 (0.1%) |
| Type 1 diabetes mellitus | 1 (0.2%) | 0 (0%) | 0 (0%) | 0 (0%) | 1 (0.1%) |
| Underweight | 1 (0.2%) | 0 (0%) | 0 (0%) | 0 (0%) | 1 (0.1%) |
| Urinary incontinence | 1 (0.2%) | 0 (0%) | 1 (0.5%) | 0 (0%) | 2 (0.2%) |
| Urinary tract infection | 2 (0.4%) | 0 (0%) | 4 (2.0%) | 1 (1.4%) | 7 (0.7%) |

| | | | | | |
|---|---|---|---|---|---|
| Urinary tract infection staphylococcal | 1 (0.2%) | 0 (0%) | 0 (0%) | 0 (0%) | 1 (0.1%) |
| Urine abnormality | 6 (1.2%) | 0 (0%) | 0 (0%) | 0 (0%) | 6 (0.6%) |
| Urine analysis abnormal | 1 (0.2%) | 0 (0%) | 0 (0%) | 0 (0%) | 1 (0.1%) |
| Urine bilirubin decreased | 1 (0.2%) | 0 (0%) | 0 (0%) | 0 (0%) | 1 (0.1%) |
| Urine bilirubin increased | 1 (0.2%) | 0 (0%) | 0 (0%) | 0 (0%) | 1 (0.1%) |
| Urine leukocyte esterase positive | 2 (0.4%) | 0 (0%) | 0 (0%) | 0 (0%) | 2 (0.2%) |
| Urticaria | 3 (0.6%) | 1 (0.4%) | 0 (0%) | 2 (2.9%) | 6 (0.6%) |
| Vaginal discharge | 1 (0.2%) | 1 (0.4%) | 0 (0%) | 0 (0%) | 2 (0.2%) |
| Vasoconstriction | 1 (0.2%) | 0 (0%) | 0 (0%) | 0 (0%) | 1 (0.1%) |
| Vein disorder | 1 (0.2%) | 0 (0%) | 0 (0%) | 0 (0%) | 1 (0.1%) |
| Vision blurred | 1 (0.2%) | 0 (0%) | 0 (0%) | 1 (1.4%) | 2 (0.2%) |
| Vomiting | 2 (0.4%) | 4 (1.6%) | 2 (1.0%) | 1 (1.4%) | 9 (0.9%) |
| Weight decreased | 3 (0.6%) | 0 (0%) | 2 (1.0%) | 2 (2.9%) | 7 (0.7%) |
| Weight increased | 2 (0.4%) | 0 (0%) | 0 (0%) | 0 (0%) | 2 (0.2%) |
| White blood cell count decreased | 2 (0.4%) | 0 (0%) | 0 (0%) | 0 (0%) | 2 (0.2%) |
| White blood cell count increased | 3 (0.6%) | 0 (0%) | 2 (1.0%) | 0 (0%) | 5 (0.5%) |
| White blood cells urine positive | 1 (0.2%) | 0 (0%) | 0 (0%) | 0 (0%) | 1 (0.1%) |
| Abdominal pain | 0 (0%) | 1 (0.4%) | 1 (0.5%) | 0 (0%) | 2 (0.2%) |
| Amenorrhoea | 0 (0%) | 1 (0.4%) | 0 (0%) | 0 (0%) | 1 (0.1%) |
| Anaphylactic reaction | 0 (0%) | 1 (0.4%) | 0 (0%) | 0 (0%) | 1 (0.1%) |
| Angioedema | 0 (0%) | 1 (0.4%) | 0 (0%) | 0 (0%) | 1 (0.1%) |
| Areflexia | 0 (0%) | 1 (0.4%) | 0 (0%) | 0 (0%) | 1 (0.1%) |
| Ataxia | 0 (0%) | 1 (0.4%) | 2 (1.0%) | 0 (0%) | 3 (0.3%) |
| Bladder hypertrophy | 0 (0%) | 1 (0.4%) | 0 (0%) | 0 (0%) | 1 (0.1%) |
| Blood pressure abnormal | 0 (0%) | 1 (0.4%) | 0 (0%) | 0 (0%) | 1 (0.1%) |
| Breast pain | 0 (0%) | 1 (0.4%) | 0 (0%) | 0 (0%) | 1 (0.1%) |
| Caesarean delivery on maternal request | 0 (0%) | 4 (1.6%) | 0 (0%) | 0 (0%) | 4 (0.4%) |
| Cardiac murmur | 0 (0%) | 1 (0.4%) | 0 (0%) | 0 (0%) | 1 (0.1%) |
| Central nervous system lesion | 0 (0%) | 2 (0.8%) | 0 (0%) | 0 (0%) | 2 (0.2%) |
| Cervix disorder | 0 (0%) | 1 (0.4%) | 0 (0%) | 0 (0%) | 1 (0.1%) |
| Chills | 0 (0%) | 1 (0.4%) | 0 (0%) | 0 (0%) | 1 (0.1%) |
| Choking | 0 (0%) | 1 (0.4%) | 0 (0%) | 0 (0%) | 1 (0.1%) |

| | | | | | |
|---|---|---|---|---|---|
| Condition aggravated | 0 (0%) | 1 (0.4%) | 0 (0%) | 0 (0%) | 1 (0.1%) |
| Congenital musculoskeletal disorder of limbs | 0 (0%) | 1 (0.4%) | 0 (0%) | 0 (0%) | 1 (0.1%) |
| Cyst | 0 (0%) | 1 (0.4%) | 0 (0%) | 0 (0%) | 1 (0.1%) |
| Deafness unilateral | 0 (0%) | 1 (0.4%) | 0 (0%) | 0 (0%) | 1 (0.1%) |
| Delivery | 0 (0%) | 1 (0.4%) | 0 (0%) | 0 (0%) | 1 (0.1%) |
| Diabetes insipidus | 0 (0%) | 1 (0.4%) | 0 (0%) | 0 (0%) | 1 (0.1%) |
| Discomfort | 0 (0%) | 1 (0.4%) | 0 (0%) | 0 (0%) | 1 (0.1%) |
| Drug hypersensitivity | 0 (0%) | 1 (0.4%) | 0 (0%) | 0 (0%) | 1 (0.1%) |
| Dysaesthesia | 0 (0%) | 1 (0.4%) | 0 (0%) | 0 (0%) | 1 (0.1%) |
| Dyskinesia | 0 (0%) | 1 (0.4%) | 0 (0%) | 0 (0%) | 1 (0.1%) |
| Expanded disability status scale score increased | 0 (0%) | 1 (0.4%) | 0 (0%) | 0 (0%) | 1 (0.1%) |
| Eye swelling | 0 (0%) | 1 (0.4%) | 0 (0%) | 0 (0%) | 1 (0.1%) |
| Eyelid sensory disorder | 0 (0%) | 1 (0.4%) | 0 (0%) | 0 (0%) | 1 (0.1%) |
| Face oedema | 0 (0%) | 1 (0.4%) | 0 (0%) | 0 (0%) | 1 (0.1%) |
| Fall | 0 (0%) | 1 (0.4%) | 1 (0.5%) | 0 (0%) | 2 (0.2%) |
| Formication | 0 (0%) | 1 (0.4%) | 0 (0%) | 0 (0%) | 1 (0.1%) |
| Gait inability | 0 (0%) | 1 (0.4%) | 0 (0%) | 0 (0%) | 1 (0.1%) |
| Gamma-glutamyltransferase increased | 0 (0%) | 1 (0.4%) | 0 (0%) | 0 (0%) | 1 (0.1%) |
| Growth retardation | 0 (0%) | 1 (0.4%) | 0 (0%) | 0 (0%) | 1 (0.1%) |
| Hand deformity | 0 (0%) | 1 (0.4%) | 0 (0%) | 0 (0%) | 1 (0.1%) |
| Heart rate decreased | 0 (0%) | 1 (0.4%) | 0 (0%) | 1 (1.4%) | 2 (0.2%) |
| Herpes simplex meningoencephalitis | 0 (0%) | 1 (0.4%) | 0 (0%) | 0 (0%) | 1 (0.1%) |
| Hormone level abnormal | 0 (0%) | 1 (0.4%) | 0 (0%) | 0 (0%) | 1 (0.1%) |
| Hyperglycaemia | 0 (0%) | 1 (0.4%) | 0 (0%) | 0 (0%) | 1 (0.1%) |
| Hyperreflexia | 0 (0%) | 1 (0.4%) | 0 (0%) | 0 (0%) | 1 (0.1%) |
| Hypersensitivity | 0 (0%) | 4 (1.6%) | 0 (0%) | 0 (0%) | 4 (0.4%) |
| Hyperthermia | 0 (0%) | 1 (0.4%) | 0 (0%) | 0 (0%) | 1 (0.1%) |
| Hypoaesthesia oral | 0 (0%) | 1 (0.4%) | 0 (0%) | 0 (0%) | 1 (0.1%) |
| Immediate post-injection reaction | 0 (0%) | 1 (0.4%) | 0 (0%) | 0 (0%) | 1 (0.1%) |
| Inadequate analgesia | 0 (0%) | 1 (0.4%) | 0 (0%) | 0 (0%) | 1 (0.1%) |
| Influenza like illness | 0 (0%) | 1 (0.4%) | 0 (0%) | 0 (0%) | 1 (0.1%) |

| | | | | | |
|---|---|---|---|---|---|
| Infusion related reaction | 0 (0%) | 1 (0.4%) | 0 (0%) | 0 (0%) | 1 (0.1%) |
| Inguinal hernia | 0 (0%) | 1 (0.4%) | 0 (0%) | 0 (0%) | 1 (0.1%) |
| Injection site atrophy | 0 (0%) | 1 (0.4%) | 0 (0%) | 0 (0%) | 1 (0.1%) |
| Injection site discolouration | 0 (0%) | 1 (0.4%) | 0 (0%) | 0 (0%) | 1 (0.1%) |
| Injection site erythema | 0 (0%) | 9 (3.5%) | 0 (0%) | 0 (0%) | 9 (0.9%) |
| Injection site haematoma | 0 (0%) | 1 (0.4%) | 0 (0%) | 0 (0%) | 1 (0.1%) |
| Injection site hypoaesthesia | 0 (0%) | 1 (0.4%) | 0 (0%) | 0 (0%) | 1 (0.1%) |
| Injection site induration | 0 (0%) | 7 (2.8%) | 0 (0%) | 0 (0%) | 7 (0.7%) |
| Injection site irritation | 0 (0%) | 1 (0.4%) | 0 (0%) | 0 (0%) | 1 (0.1%) |
| Injection site mass | 0 (0%) | 2 (0.8%) | 0 (0%) | 0 (0%) | 2 (0.2%) |
| Injection site pain | 0 (0%) | 6 (2.4%) | 0 (0%) | 0 (0%) | 6 (0.6%) |
| Injection site pruritus | 0 (0%) | 5 (2.0%) | 0 (0%) | 0 (0%) | 5 (0.5%) |
| Injection site swelling | 0 (0%) | 4 (1.6%) | 0 (0%) | 0 (0%) | 4 (0.4%) |
| Injection site urticaria | 0 (0%) | 1 (0.4%) | 0 (0%) | 0 (0%) | 1 (0.1%) |
| Interferon gamma release assay positive | 0 (0%) | 1 (0.4%) | 0 (0%) | 0 (0%) | 1 (0.1%) |
| Iron deficiency anaemia | 0 (0%) | 1 (0.4%) | 0 (0%) | 0 (0%) | 1 (0.1%) |
| JC virus infection | 0 (0%) | 1 (0.4%) | 0 (0%) | 0 (0%) | 1 (0.1%) |
| Lactation disorder | 0 (0%) | 1 (0.4%) | 0 (0%) | 0 (0%) | 1 (0.1%) |
| Lhermitte's sign | 0 (0%) | 1 (0.4%) | 0 (0%) | 0 (0%) | 1 (0.1%) |
| Lip swelling | 0 (0%) | 1 (0.4%) | 0 (0%) | 0 (0%) | 1 (0.1%) |
| Lymphadenopathy | 0 (0%) | 1 (0.4%) | 0 (0%) | 0 (0%) | 1 (0.1%) |
| Lymphocytosis | 0 (0%) | 1 (0.4%) | 0 (0%) | 0 (0%) | 1 (0.1%) |
| Magnetic resonance imaging abnormal | 0 (0%) | 1 (0.4%) | 0 (0%) | 1 (1.4%) | 2 (0.2%) |
| Menometrorrhagia | 0 (0%) | 1 (0.4%) | 0 (0%) | 0 (0%) | 1 (0.1%) |
| Movement disorder | 0 (0%) | 1 (0.4%) | 0 (0%) | 0 (0%) | 1 (0.1%) |
| Needle fatigue | 0 (0%) | 1 (0.4%) | 0 (0%) | 0 (0%) | 1 (0.1%) |
| Obesity | 0 (0%) | 1 (0.4%) | 0 (0%) | 0 (0%) | 1 (0.1%) |
| Overweight | 0 (0%) | 1 (0.4%) | 0 (0%) | 0 (0%) | 1 (0.1%) |
| Paraesthesia | 0 (0%) | 2 (0.8%) | 0 (0%) | 0 (0%) | 2 (0.2%) |
| Perinatal depression | 0 (0%) | 1 (0.4%) | 1 (0.5%) | 0 (0%) | 2 (0.2%) |
| Post procedural discomfort | 0 (0%) | 1 (0.4%) | 0 (0%) | 0 (0%) | 1 (0.1%) |
| Presyncope | 0 (0%) | 1 (0.4%) | 0 (0%) | 0 (0%) | 1 (0.1%) |

| | | | | | |
|---|---|---|---|---|---|
| Procedural pain | 0 (0%) | 1 (0.4%) | 0 (0%) | 0 (0%) | 1 (0.1%) |
| Progesterone decreased | 0 (0%) | 1 (0.4%) | 0 (0%) | 0 (0%) | 1 (0.1%) |
| Proteinuria | 0 (0%) | 1 (0.4%) | 0 (0%) | 0 (0%) | 1 (0.1%) |
| Salpingectomy | 0 (0%) | 1 (0.4%) | 0 (0%) | 0 (0%) | 1 (0.1%) |
| Self-consciousness | 0 (0%) | 1 (0.4%) | 0 (0%) | 0 (0%) | 1 (0.1%) |
| Sense of oppression | 0 (0%) | 1 (0.4%) | 0 (0%) | 0 (0%) | 1 (0.1%) |
| Sepsis | 0 (0%) | 1 (0.4%) | 2 (1.0%) | 0 (0%) | 3 (0.3%) |
| Skin indentation | 0 (0%) | 1 (0.4%) | 0 (0%) | 0 (0%) | 1 (0.1%) |
| Skin reaction | 0 (0%) | 1 (0.4%) | 0 (0%) | 0 (0%) | 1 (0.1%) |
| Skin striae | 0 (0%) | 1 (0.4%) | 0 (0%) | 0 (0%) | 1 (0.1%) |
| Spinal cord disorder | 0 (0%) | 1 (0.4%) | 0 (0%) | 0 (0%) | 1 (0.1%) |
| Stress | 0 (0%) | 2 (0.8%) | 1 (0.5%) | 0 (0%) | 3 (0.3%) |
| Suppressed lactation | 0 (0%) | 3 (1.2%) | 0 (0%) | 0 (0%) | 3 (0.3%) |
| Supraventricular tachycardia | 0 (0%) | 2 (0.8%) | 0 (0%) | 0 (0%) | 2 (0.2%) |
| Swelling | 0 (0%) | 2 (0.8%) | 0 (0%) | 0 (0%) | 2 (0.2%) |
| Syncope | 0 (0%) | 3 (1.2%) | 0 (0%) | 0 (0%) | 3 (0.3%) |
| Therapeutic product effect incomplete | 0 (0%) | 1 (0.4%) | 1 (0.5%) | 0 (0%) | 2 (0.2%) |
| Therapeutic product effect variable | 0 (0%) | 1 (0.4%) | 0 (0%) | 0 (0%) | 1 (0.1%) |
| Throat tightness | 0 (0%) | 1 (0.4%) | 0 (0%) | 1 (1.4%) | 2 (0.2%) |
| Umbilical hernia | 0 (0%) | 1 (0.4%) | 0 (0%) | 0 (0%) | 1 (0.1%) |
| Uterine disorder | 0 (0%) | 1 (0.4%) | 0 (0%) | 0 (0%) | 1 (0.1%) |
| Vaginal haemorrhage | 0 (0%) | 1 (0.4%) | 2 (1.0%) | 3 (4.3%) | 6 (0.6%) |
| Vertigo positional | 0 (0%) | 1 (0.4%) | 0 (0%) | 0 (0%) | 1 (0.1%) |
| Visual impairment | 0 (0%) | 2 (0.8%) | 0 (0%) | 0 (0%) | 2 (0.2%) |
| Wheezing | 0 (0%) | 1 (0.4%) | 0 (0%) | 0 (0%) | 1 (0.1%) |
| White matter lesion | 0 (0%) | 1 (0.4%) | 0 (0%) | 0 (0%) | 1 (0.1%) |
| Arrhythmia | 0 (0%) | 0 (0%) | 1 (0.5%) | 0 (0%) | 1 (0.1%) |
| Autism spectrum disorder | 0 (0%) | 0 (0%) | 1 (0.5%) | 0 (0%) | 1 (0.1%) |
| Autoimmune demyelinating disease | 0 (0%) | 0 (0%) | 1 (0.5%) | 0 (0%) | 1 (0.1%) |
| Autoimmune haemolytic anaemia | 0 (0%) | 0 (0%) | 1 (0.5%) | 0 (0%) | 1 (0.1%) |
| Autoimmune neutropenia | 0 (0%) | 0 (0%) | 1 (0.5%) | 0 (0%) | 1 (0.1%) |
| Autonomic nervous system imbalance | 0 (0%) | 0 (0%) | 1 (0.5%) | 0 (0%) | 1 (0.1%) |

| | | | | | |
|---|---|---|---|---|---|
| Bacterial sepsis | 0 (0%) | 0 (0%) | 1 (0.5%) | 0 (0%) | 1 (0.1%) |
| Beta haemolytic streptococcal infection | 0 (0%) | 0 (0%) | 1 (0.5%) | 0 (0%) | 1 (0.1%) |
| Blood bilirubin increased | 0 (0%) | 0 (0%) | 1 (0.5%) | 0 (0%) | 1 (0.1%) |
| Cardiogenic shock | 0 (0%) | 0 (0%) | 1 (0.5%) | 0 (0%) | 1 (0.1%) |
| Cervix haematoma uterine | 0 (0%) | 0 (0%) | 1 (0.5%) | 0 (0%) | 1 (0.1%) |
| Cervix haemorrhage uterine | 0 (0%) | 0 (0%) | 1 (0.5%) | 0 (0%) | 1 (0.1%) |
| Coagulopathy | 0 (0%) | 0 (0%) | 1 (0.5%) | 0 (0%) | 1 (0.1%) |
| Coronavirus infection | 0 (0%) | 0 (0%) | 1 (0.5%) | 0 (0%) | 1 (0.1%) |
| COVID-19 | 0 (0%) | 0 (0%) | 2 (1.0%) | 2 (2.9%) | 4 (0.4%) |
| Coxsackie myocarditis | 0 (0%) | 0 (0%) | 1 (0.5%) | 0 (0%) | 1 (0.1%) |
| Coxsackie viral infection | 0 (0%) | 0 (0%) | 1 (0.5%) | 0 (0%) | 1 (0.1%) |
| Dehydration | 0 (0%) | 0 (0%) | 1 (0.5%) | 0 (0%) | 1 (0.1%) |
| Depressed level of consciousness | 0 (0%) | 0 (0%) | 1 (0.5%) | 0 (0%) | 1 (0.1%) |
| Diaphragmatic injury | 0 (0%) | 0 (0%) | 1 (0.5%) | 0 (0%) | 1 (0.1%) |
| Drug dependence | 0 (0%) | 0 (0%) | 1 (0.5%) | 0 (0%) | 1 (0.1%) |
| Dysmetria | 0 (0%) | 0 (0%) | 1 (0.5%) | 0 (0%) | 1 (0.1%) |
| Epilepsy | 0 (0%) | 0 (0%) | 1 (0.5%) | 0 (0%) | 1 (0.1%) |
| Escherichia vaginitis | 0 (0%) | 0 (0%) | 1 (0.5%) | 0 (0%) | 1 (0.1%) |
| Female reproductive tract disorder | 0 (0%) | 0 (0%) | 1 (0.5%) | 0 (0%) | 1 (0.1%) |
| Gastritis | 0 (0%) | 0 (0%) | 1 (0.5%) | 0 (0%) | 1 (0.1%) |
| Gastrointestinal disorder | 0 (0%) | 0 (0%) | 1 (0.5%) | 0 (0%) | 1 (0.1%) |
| General physical health deterioration | 0 (0%) | 0 (0%) | 1 (0.5%) | 0 (0%) | 1 (0.1%) |
| Hepatic enzyme increased | 0 (0%) | 0 (0%) | 1 (0.5%) | 0 (0%) | 1 (0.1%) |
| Human chorionic gonadotropin decreased | 0 (0%) | 0 (0%) | 1 (0.5%) | 0 (0%) | 1 (0.1%) |
| Hydrocephalus | 0 (0%) | 0 (0%) | 2 (1.0%) | 0 (0%) | 2 (0.2%) |
| Hydronephrosis | 0 (0%) | 0 (0%) | 2 (1.0%) | 1 (1.4%) | 3 (0.3%) |
| Hyperbilirubinaemia | 0 (0%) | 0 (0%) | 1 (0.5%) | 0 (0%) | 1 (0.1%) |
| Hypotonia | 0 (0%) | 0 (0%) | 1 (0.5%) | 0 (0%) | 1 (0.1%) |
| Infertility | 0 (0%) | 0 (0%) | 1 (0.5%) | 0 (0%) | 1 (0.1%) |
| Intention tremor | 0 (0%) | 0 (0%) | 1 (0.5%) | 0 (0%) | 1 (0.1%) |
| Iron deficiency | 0 (0%) | 0 (0%) | 1 (0.5%) | 0 (0%) | 1 (0.1%) |
| Kidney enlargement | 0 (0%) | 0 (0%) | 1 (0.5%) | 0 (0%) | 1 (0.1%) |

| | | | | | |
|---|---|---|---|---|---|
| Kidney infection | 0 (0%) | 0 (0%) | 1 (0.5%) | 0 (0%) | 1 (0.1%) |
| Leukocytosis | 0 (0%) | 0 (0%) | 2 (1.0%) | 0 (0%) | 2 (0.2%) |
| Ligament sprain | 0 (0%) | 0 (0%) | 1 (0.5%) | 0 (0%) | 1 (0.1%) |
| Liver function test increased | 0 (0%) | 0 (0%) | 1 (0.5%) | 0 (0%) | 1 (0.1%) |
| Lymphocyte count increased | 0 (0%) | 0 (0%) | 1 (0.5%) | 0 (0%) | 1 (0.1%) |
| Mastitis | 0 (0%) | 0 (0%) | 1 (0.5%) | 0 (0%) | 1 (0.1%) |
| Mean cell haemoglobin concentration decreased | 0 (0%) | 0 (0%) | 1 (0.5%) | 0 (0%) | 1 (0.1%) |
| Meningitis coxsackie viral | 0 (0%) | 0 (0%) | 1 (0.5%) | 0 (0%) | 1 (0.1%) |
| Meningitis enteroviral | 0 (0%) | 0 (0%) | 1 (0.5%) | 0 (0%) | 1 (0.1%) |
| Mitral valve incompetence | 0 (0%) | 0 (0%) | 1 (0.5%) | 0 (0%) | 1 (0.1%) |
| Myelitis | 0 (0%) | 0 (0%) | 1 (0.5%) | 0 (0%) | 1 (0.1%) |
| Neck pain | 0 (0%) | 0 (0%) | 1 (0.5%) | 0 (0%) | 1 (0.1%) |
| Neoplasm malignant | 0 (0%) | 0 (0%) | 1 (0.5%) | 0 (0%) | 1 (0.1%) |
| Nephrolithiasis | 0 (0%) | 0 (0%) | 1 (0.5%) | 0 (0%) | 1 (0.1%) |
| Nephropathy | 0 (0%) | 0 (0%) | 1 (0.5%) | 0 (0%) | 1 (0.1%) |
| Neurological decompensation | 0 (0%) | 0 (0%) | 3 (1.5%) | 0 (0%) | 3 (0.3%) |
| Oedema | 0 (0%) | 0 (0%) | 2 (1.0%) | 0 (0%) | 2 (0.2%) |
| Optic neuritis | 0 (0%) | 0 (0%) | 3 (1.5%) | 0 (0%) | 3 (0.3%) |
| Paraparesis | 0 (0%) | 0 (0%) | 1 (0.5%) | 0 (0%) | 1 (0.1%) |
| Pelvic deformity | 0 (0%) | 0 (0%) | 1 (0.5%) | 0 (0%) | 1 (0.1%) |
| Platelet count abnormal | 0 (0%) | 0 (0%) | 1 (0.5%) | 0 (0%) | 1 (0.1%) |
| Pleural effusion | 0 (0%) | 0 (0%) | 1 (0.5%) | 0 (0%) | 1 (0.1%) |
| Post procedural haematoma | 0 (0%) | 0 (0%) | 1 (0.5%) | 0 (0%) | 1 (0.1%) |
| Protein total decreased | 0 (0%) | 0 (0%) | 1 (0.5%) | 0 (0%) | 1 (0.1%) |
| Renal cyst | 0 (0%) | 0 (0%) | 1 (0.5%) | 0 (0%) | 1 (0.1%) |
| Renal failure | 0 (0%) | 0 (0%) | 1 (0.5%) | 0 (0%) | 1 (0.1%) |
| Respiratory failure | 0 (0%) | 0 (0%) | 1 (0.5%) | 0 (0%) | 1 (0.1%) |
| Respiratory tract infection viral | 0 (0%) | 0 (0%) | 1 (0.5%) | 0 (0%) | 1 (0.1%) |
| SARS-CoV-2 test positive | 0 (0%) | 0 (0%) | 1 (0.5%) | 1 (1.4%) | 2 (0.2%) |
| Selective eating disorder | 0 (0%) | 0 (0%) | 1 (0.5%) | 0 (0%) | 1 (0.1%) |
| Spinal pain | 0 (0%) | 0 (0%) | 1 (0.5%) | 0 (0%) | 1 (0.1%) |

| | | | | | |
|---|---|---|---|---|---|
| Strabismus | 0 (0%) | 0 (0%) | 1 (0.5%) | 0 (0%) | 1 (0.1%) |
| Suture rupture | 0 (0%) | 0 (0%) | 1 (0.5%) | 0 (0%) | 1 (0.1%) |
| Symphysiolysis | 0 (0%) | 0 (0%) | 1 (0.5%) | 0 (0%) | 1 (0.1%) |
| Toxoplasmosis | 0 (0%) | 0 (0%) | 1 (0.5%) | 0 (0%) | 1 (0.1%) |
| Trigeminal neuralgia | 0 (0%) | 0 (0%) | 1 (0.5%) | 0 (0%) | 1 (0.1%) |
| Uterine cervical laceration | 0 (0%) | 0 (0%) | 1 (0.5%) | 0 (0%) | 1 (0.1%) |
| Uterine leiomyoma | 0 (0%) | 0 (0%) | 1 (0.5%) | 0 (0%) | 1 (0.1%) |
| Vaginal infection | 0 (0%) | 0 (0%) | 1 (0.5%) | 0 (0%) | 1 (0.1%) |
| Vaginal perforation | 0 (0%) | 0 (0%) | 1 (0.5%) | 0 (0%) | 1 (0.1%) |
| Vertigo | 0 (0%) | 0 (0%) | 1 (0.5%) | 0 (0%) | 1 (0.1%) |
| Viral myocarditis | 0 (0%) | 0 (0%) | 1 (0.5%) | 0 (0%) | 1 (0.1%) |
| Weight abnormal | 0 (0%) | 0 (0%) | 1 (0.5%) | 0 (0%) | 1 (0.1%) |
| Wolff-Parkinson-White syndrome | 0 (0%) | 0 (0%) | 1 (0.5%) | 0 (0%) | 1 (0.1%) |
| B-lymphocyte count decreased | 0 (0%) | 0 (0%) | 0 (0%) | 1 (1.4%) | 1 (0.1%) |
| Blindness unilateral | 0 (0%) | 0 (0%) | 0 (0%) | 1 (1.4%) | 1 (0.1%) |
| Burning sensation | 0 (0%) | 0 (0%) | 0 (0%) | 1 (1.4%) | 1 (0.1%) |
| COVID-19 pneumonia | 0 (0%) | 0 (0%) | 0 (0%) | 2 (2.9%) | 2 (0.2%) |
| Cystitis bacterial | 0 (0%) | 0 (0%) | 0 (0%) | 1 (1.4%) | 1 (0.1%) |
| Decreased embryo viability | 0 (0%) | 0 (0%) | 0 (0%) | 1 (1.4%) | 1 (0.1%) |
| Desmoid tumour | 0 (0%) | 0 (0%) | 0 (0%) | 1 (1.4%) | 1 (0.1%) |
| Dysphagia | 0 (0%) | 0 (0%) | 0 (0%) | 1 (1.4%) | 1 (0.1%) |
| Erythema multiforme | 0 (0%) | 0 (0%) | 0 (0%) | 1 (1.4%) | 1 (0.1%) |
| Fungal infection | 0 (0%) | 0 (0%) | 0 (0%) | 1 (1.4%) | 1 (0.1%) |
| Immunoglobulins decreased | 0 (0%) | 0 (0%) | 0 (0%) | 1 (1.4%) | 1 (0.1%) |
| Injury | 0 (0%) | 0 (0%) | 0 (0%) | 1 (1.4%) | 1 (0.1%) |
| Jaundice | 0 (0%) | 0 (0%) | 0 (0%) | 2 (2.9%) | 2 (0.2%) |
| Liver function test abnormal | 0 (0%) | 0 (0%) | 0 (0%) | 1 (1.4%) | 1 (0.1%) |
| Macrosomia | 0 (0%) | 0 (0%) | 0 (0%) | 1 (1.4%) | 1 (0.1%) |
| Muscle spasticity | 0 (0%) | 0 (0%) | 0 (0%) | 1 (1.4%) | 1 (0.1%) |
| Muscle twitching | 0 (0%) | 0 (0%) | 0 (0%) | 1 (1.4%) | 1 (0.1%) |
| Neuromyelitis optica spectrum disorder | 0 (0%) | 0 (0%) | 0 (0%) | 1 (1.4%) | 1 (0.1%) |
| Pneumonia bacterial | 0 (0%) | 0 (0%) | 0 (0%) | 1 (1.4%) | 1 (0.1%) |

| | | | | | |
|---|---|---|---|---|---|
| Psoriasis | 0 (0%) | 0 (0%) | 0 (0%) | 1 (1.4%) | 1 (0.1%) |
| Seizure | 0 (0%) | 0 (0%) | 0 (0%) | 1 (1.4%) | 1 (0.1%) |
| Somatic symptom disorder of pregnancy | 0 (0%) | 0 (0%) | 0 (0%) | 1 (1.4%) | 1 (0.1%) |
| Swelling of eyelid | 0 (0%) | 0 (0%) | 0 (0%) | 1 (1.4%) | 1 (0.1%) |
| Throat irritation | 0 (0%) | 0 (0%) | 0 (0%) | 1 (1.4%) | 1 (0.1%) |

## Part II – Termination of pregnancy and risk of abortion

| Termination of pregnancy and risk of abortion | ALEMTUZUMAB (N=20) | GLATIRAMER (N=55) | NATALIZUMAB (N=266) | OCRELIZUMAB (N=38) | Overall (N=379) |
|---|---|---|---|---|---|
| Abortion | 2 (10.0%) | 6 (10.9%) | 8 (3.0%) | 0 (0%) | 16 (4.2%) |
| Abortion incomplete | 3 (15.0%) | 0 (0%) | 0 (0%) | 0 (0%) | 3 (0.8%) |
| Abortion induced | 0 (0%) | 2 (3.6%) | 4 (1.5%) | 0 (0%) | 6 (1.6%) |
| Abortion missed | 0 (0%) | 3 (5.5%) | 6 (2.3%) | 3 (7.9%) | 12 (3.2%) |
| Abortion of ectopic pregnancy | 0 (0%) | 0 (0%) | 1 (0.4%) | 0 (0%) | 1 (0.3%) |
| Abortion spontaneous | 13 (65.0%) | 42 (76.4%) | 235 (88.3%) | 31 (81.6%) | 321 (84.7%) |
| Abortion spontaneous complete | 1 (5.0%) | 0 (0%) | 0 (0%) | 0 (0%) | 1 (0.3%) |
| Abortion threatened | 0 (0%) | 0 (0%) | 3 (1.1%) | 0 (0%) | 3 (0.8%) |
| Anembryonic gestation | 0 (0%) | 1 (1.8%) | 2 (0.8%) | 0 (0%) | 3 (0.8%) |
| Foetal death | 1 (5.0%) | 1 (1.8%) | 2 (0.8%) | 2 (5.3%) | 6 (1.6%) |
| Habitual abortion | 0 (0%) | 0 (0%) | 0 (0%) | 1 (2.6%) | 1 (0.3%) |
| Stillbirth | 0 (0%) | 0 (0%) | 5 (1.9%) | 1 (2.6%) | 6 (1.6%) |

**Part III - Pregnancy, labour and delivery complications and risk factors (excl abortions and stillbirth)**

| Pregnancy, labour and delivery complications and risk factors (excl abortions and stillbirth) | ALEMTUZUMAB (N=7) | GLATIRAMER (N=54) | NATALIZUMAB (N=284) | OCRELIZUMAB (N=15) | Overall (N=360) |
|---|---|---|---|---|---|
| Abnormal labour | 0 (0%) | 0 (0%) | 1 (0.1%) | 0 (0%) | 1 (0.3%) |
| Amniotic cavity infection | 0 (0%) | 0 (0%) | 3 (0.3%) | 1 (1.0%) | 4 (1.1%) |
| Amniotic fluid volume decreased | 0 (0%) | 2 (0.8%) | 2 (0.2%) | 0 (0%) | 4 (1.1%) |
| Anaemia of pregnancy | 0 (0%) | 0 (0%) | 2 (0.2%) | 0 (0%) | 2 (0.6%) |
| Assisted delivery | 0 (0%) | 0 (0%) | 1 (0.1%) | 0 (0%) | 1 (0.3%) |
| Bacteriuria in pregnancy | 0 (0%) | 0 (0%) | 1 (0.1%) | 0 (0%) | 1 (0.3%) |
| Benign hydatidiform mole | 0 (0%) | 0 (0%) | 1 (0.1%) | 0 (0%) | 1 (0.3%) |
| Breech presentation | 0 (0%) | 1 (0.4%) | 7 (0.7%) | 0 (0%) | 8 (2.2%) |
| Caesarean section | 0 (0%) | 13 (5.5%) | 93 (8.8%) | 0 (0%) | 106 (29.5%) |
| Cephalo-pelvic disproportion | 0 (0%) | 1 (0.4%) | 0 (0%) | 0 (0%) | 1 (0.3%) |
| Cervical incompetence | 1 (1.4%) | 1 (0.4%) | 4 (0.4%) | 0 (0%) | 6 (1.7%) |
| Cervix dystocia | 0 (0%) | 0 (0%) | 2 (0.2%) | 0 (0%) | 2 (0.6%) |
| Cholestasis of pregnancy | 0 (0%) | 0 (0%) | 4 (0.4%) | 0 (0%) | 4 (1.1%) |
| Complication of delivery | 0 (0%) | 1 (0.4%) | 0 (0%) | 0 (0%) | 1 (0.3%) |
| Complication of pregnancy | 0 (0%) | 0 (0%) | 3 (0.3%) | 0 (0%) | 3 (0.8%) |
| Ectopic pregnancy | 2 (2.8%) | 3 (1.3%) | 9 (0.9%) | 0 (0%) | 14 (3.9%) |
| Face presentation | 0 (0%) | 0 (0%) | 1 (0.1%) | 0 (0%) | 1 (0.3%) |
| Failed induction of labour | 0 (0%) | 0 (0%) | 1 (0.1%) | 0 (0%) | 1 (0.3%) |
| Foetal malposition | 0 (0%) | 1 (0.4%) | 5 (0.5%) | 0 (0%) | 6 (1.7%) |
| Foetal malpresentation | 0 (0%) | 0 (0%) | 1 (0.1%) | 0 (0%) | 1 (0.3%) |
| Gestational diabetes | 1 (1.4%) | 6 (2.5%) | 8 (0.8%) | 3 (2.9%) | 18 (5.0%) |
| Gestational hypertension | 0 (0%) | 1 (0.4%) | 2 (0.2%) | 0 (0%) | 3 (0.8%) |
| Haemorrhage in pregnancy | 0 (0%) | 2 (0.8%) | 8 (0.8%) | 0 (0%) | 10 (2.8%) |
| HELLP syndrome | 0 (0%) | 0 (0%) | 3 (0.3%) | 0 (0%) | 3 (0.8%) |
| High risk pregnancy | 0 (0%) | 0 (0%) | 1 (0.1%) | 0 (0%) | 1 (0.3%) |

| | | | | | |
|---|---|---|---|---|---|
| Hyperemesis gravidarum | 0 (0%) | 2 (0.8%) | 4 (0.4%) | 0 (0%) | 6 (1.7%) |
| Intrapartum haemorrhage | 0 (0%) | 0 (0%) | 1 (0.1%) | 0 (0%) | 1 (0.3%) |
| Labour complication | 0 (0%) | 1 (0.4%) | 0 (0%) | 0 (0%) | 1 (0.3%) |
| Large for dates baby | 0 (0%) | 1 (0.4%) | 2 (0.2%) | 0 (0%) | 3 (0.8%) |
| Morning sickness | 2 (2.8%) | 1 (0.4%) | 0 (0%) | 0 (0%) | 3 (0.8%) |
| Oblique presentation | 0 (0%) | 0 (0%) | 1 (0.1%) | 0 (0%) | 1 (0.3%) |
| Obstructed labour | 0 (0%) | 0 (0%) | 1 (0.1%) | 0 (0%) | 1 (0.3%) |
| Oligohydramnios | 0 (0%) | 1 (0.4%) | 2 (0.2%) | 1 (1.0%) | 4 (1.1%) |
| Perineal injury | 0 (0%) | 0 (0%) | 4 (0.4%) | 0 (0%) | 4 (1.1%) |
| Placenta praevia | 0 (0%) | 0 (0%) | 2 (0.2%) | 0 (0%) | 2 (0.6%) |
| Placental disorder | 0 (0%) | 0 (0%) | 3 (0.3%) | 0 (0%) | 3 (0.8%) |
| Placental infarction | 0 (0%) | 0 (0%) | 1 (0.1%) | 0 (0%) | 1 (0.3%) |
| Placental insufficiency | 0 (0%) | 0 (0%) | 3 (0.3%) | 0 (0%) | 3 (0.8%) |
| Placental transfusion syndrome | 0 (0%) | 0 (0%) | 3 (0.3%) | 0 (0%) | 3 (0.8%) |
| Polyhydramnios | 0 (0%) | 0 (0%) | 0 (0%) | 1 (1.0%) | 1 (0.3%) |
| Polymorphic eruption of pregnancy | 0 (0%) | 1 (0.4%) | 0 (0%) | 0 (0%) | 1 (0.3%) |
| Postpartum haemorrhage | 0 (0%) | 0 (0%) | 2 (0.2%) | 0 (0%) | 2 (0.6%) |
| Precipitate labour | 0 (0%) | 0 (0%) | 1 (0.1%) | 0 (0%) | 1 (0.3%) |
| Pre-eclampsia | 0 (0%) | 1 (0.4%) | 16 (1.5%) | 0 (0%) | 17 (4.7%) |
| Pregnancy with advanced maternal age | 0 (0%) | 0 (0%) | 1 (0.1%) | 0 (0%) | 1 (0.3%) |
| Premature delivery | 0 (0%) | 2 (0.8%) | 35 (3.3%) | 3 (2.9%) | 40 (11.1%) |
| Premature labour | 0 (0%) | 4 (1.7%) | 5 (0.5%) | 1 (1.0%) | 10 (2.8%) |
| Premature rupture of membranes | 0 (0%) | 1 (0.4%) | 6 (0.6%) | 1 (1.0%) | 8 (2.2%) |
| Premature separation of placenta | 0 (0%) | 0 (0%) | 5 (0.5%) | 0 (0%) | 5 (1.4%) |
| Preterm premature rupture of membranes | 0 (0%) | 1 (0.4%) | 1 (0.1%) | 0 (0%) | 2 (0.6%) |
| Prolonged labour | 0 (0%) | 3 (1.3%) | 7 (0.7%) | 0 (0%) | 10 (2.8%) |
| Prolonged pregnancy | 0 (0%) | 0 (0%) | 1 (0.1%) | 0 (0%) | 1 (0.3%) |
| Retained placenta or membranes | 0 (0%) | 1 (0.4%) | 0 (0%) | 0 (0%) | 1 (0.3%) |
| Small size placenta | 0 (0%) | 0 (0%) | 2 (0.2%) | 0 (0%) | 2 (0.6%) |

| | | | | | |
|---|---|---|---|---|---|
| Subchorionic haematoma | 0 (0%) | 0 (0%) | 1 (0.1%) | 0 (0%) | 1 (0.3%) |
| Transverse presentation | 0 (0%) | 0 (0%) | 1 (0.1%) | 0 (0%) | 1 (0.3%) |
| Twin pregnancy | 0 (0%) | 0 (0%) | 1 (0.1%) | 2 (2.0%) | 3 (0.8%) |
| Umbilical cord abnormality | 0 (0%) | 1 (0.4%) | 2 (0.2%) | 2 (2.0%) | 5 (1.4%) |
| Umbilical cord around neck | 1 (1.4%) | 0 (0%) | 2 (0.2%) | 0 (0%) | 3 (0.8%) |
| Umbilical cord cyst | 0 (0%) | 0 (0%) | 1 (0.1%) | 0 (0%) | 1 (0.3%) |
| Uterine contractions abnormal | 0 (0%) | 1 (0.4%) | 0 (0%) | 0 (0%) | 1 (0.3%) |
| Uterine hypotonus | 0 (0%) | 0 (0%) | 1 (0.1%) | 0 (0%) | 1 (0.3%) |
| Uterine rupture | 0 (0%) | 0 (0%) | 1 (0.1%) | 0 (0%) | 1 (0.3%) |
| Vulvovaginal injury | 0 (0%) | 0 (0%) | 2 (0.2%) | 0 (0%) | 2 (0.6%) |

**Part IV - Neonatal disorders**

| Neonatal disorders | ALEMTUZUMAB (N=8) | GLATIRAMER (N=18) | NATALIZUMAB (N=130) | OCRELIZUMAB (N=6) | Overall (N=162) |
|---|---|---|---|---|---|
| Anaemia neonatal | 0 (0%) | 0 (0%) | 16 (12.3%) | 1 (16.7%) | 17 (10.5%) |
| Apgar score low | 0 (0%) | 1 (5.6%) | 0 (0%) | 0 (0%) | 1 (0.6%) |
| Arrhythmia neonatal | 0 (0%) | 1 (5.6%) | 1 (0.8%) | 0 (0%) | 2 (1.2%) |
| Cyanosis neonatal | 0 (0%) | 0 (0%) | 1 (0.8%) | 0 (0%) | 1 (0.6%) |
| Death neonatal | 0 (0%) | 0 (0%) | 3 (2.3%) | 0 (0%) | 3 (1.9%) |
| Disturbance of thermoregulation of newborn | 0 (0%) | 0 (0%) | 1 (0.8%) | 0 (0%) | 1 (0.6%) |
| Fever neonatal | 0 (0%) | 0 (0%) | 1 (0.8%) | 0 (0%) | 1 (0.6%) |
| Hypoglycaemia neonatal | 1 (12.5%) | 1 (5.6%) | 1 (0.8%) | 0 (0%) | 3 (1.9%) |

| | | | | | |
|---|---|---|---|---|---|
| Hypotonia neonatal | 1 (12.5%) | 0 (0%) | 0 (0%) | 0 (0%) | 1 (0.6%) |
| Infantile apnoea | 0 (0%) | 0 (0%) | 1 (0.8%) | 0 (0%) | 1 (0.6%) |
| Infantile haemangioma | 0 (0%) | 1 (5.6%) | 0 (0%) | 0 (0%) | 1 (0.6%) |
| Intraventricular haemorrhage neonatal | 1 (12.5%) | 0 (0%) | 0 (0%) | 0 (0%) | 1 (0.6%) |
| Jaundice neonatal | 0 (0%) | 1 (5.6%) | 5 (3.8%) | 0 (0%) | 6 (3.7%) |
| Low birth weight baby | 0 (0%) | 1 (5.6%) | 24 (18.5%) | 0 (0%) | 25 (15.4%) |
| Maternal exposure during delivery | 0 (0%) | 1 (5.6%) | 0 (0%) | 0 (0%) | 1 (0.6%) |
| Meningitis neonatal | 0 (0%) | 0 (0%) | 3 (2.3%) | 0 (0%) | 3 (1.9%) |
| Myasthenia gravis neonatal | 2 (25.0%) | 0 (0%) | 0 (0%) | 0 (0%) | 2 (1.2%) |
| Neonatal anoxia | 0 (0%) | 1 (5.6%) | 0 (0%) | 0 (0%) | 1 (0.6%) |
| Neonatal asphyxia | 0 (0%) | 0 (0%) | 1 (0.8%) | 0 (0%) | 1 (0.6%) |
| Neonatal behavioural syndrome | 1 (12.5%) | 0 (0%) | 0 (0%) | 0 (0%) | 1 (0.6%) |
| Neonatal cardiac failure | 0 (0%) | 0 (0%) | 1 (0.8%) | 0 (0%) | 1 (0.6%) |
| Neonatal gastrointestinal disorder | 0 (0%) | 0 (0%) | 1 (0.8%) | 0 (0%) | 1 (0.6%) |
| Neonatal hypoacusis | 0 (0%) | 0 (0%) | 1 (0.8%) | 0 (0%) | 1 (0.6%) |
| Neonatal hypoxia | 0 (0%) | 1 (5.6%) | 0 (0%) | 0 (0%) | 1 (0.6%) |
| Neonatal infection | 0 (0%) | 0 (0%) | 2 (1.5%) | 0 (0%) | 2 (1.2%) |
| Neonatal multi-organ failure | 0 (0%) | 0 (0%) | 1 (0.8%) | 0 (0%) | 1 (0.6%) |
| Neonatal pneumothorax | 0 (0%) | 0 (0%) | 0 (0%) | 1 (16.7%) | 1 (0.6%) |
| Neonatal respiratory distress | 0 (0%) | 1 (5.6%) | 1 (0.8%) | 0 (0%) | 2 (1.2%) |
| Neonatal respiratory distress syndrome | 0 (0%) | 0 (0%) | 1 (0.8%) | 0 (0%) | 1 (0.6%) |
| Neonatal respiratory failure | 0 (0%) | 0 (0%) | 1 (0.8%) | 0 (0%) | 1 (0.6%) |
| Neonatal thyrotoxicosis | 1 (12.5%) | 0 (0%) | 0 (0%) | 0 (0%) | 1 (0.6%) |
| Perinatal brain damage | 0 (0%) | 0 (0%) | 1 (0.8%) | 0 (0%) | 1 (0.6%) |
| Poor feeding infant | 0 (0%) | 0 (0%) | 2 (1.5%) | 0 (0%) | 2 (1.2%) |
| Poor sucking reflex | 0 (0%) | 0 (0%) | 2 (1.5%) | 0 (0%) | 2 (1.2%) |
| Poor weight gain neonatal | 0 (0%) | 1 (5.6%) | 0 (0%) | 0 (0%) | 1 (0.6%) |
| Premature baby | 1 (12.5%) | 5 (27.8%) | 52 (40.0%) | 4 (66.7%) | 62 (38.3%) |
| Pulmonary oedema neonatal | 0 (0%) | 0 (0%) | 1 (0.8%) | 0 (0%) | 1 (0.6%) |

| | | | | | |
|---|---|---|---|---|---|
| Respiratory disorder neonatal | 0 (0%) | 0 (0%) | 1 (0.8%) | 0 (0%) | 1 (0.6%) |
| Sepsis neonatal | 0 (0%) | 1 (5.6%) | 0 (0%) | 0 (0%) | 1 (0.6%) |
| Small for dates baby | 0 (0%) | 1 (5.6%) | 2 (1.5%) | 0 (0%) | 3 (1.9%) |
| Thrombocytopenia neonatal | 0 (0%) | 0 (0%) | 2 (1.5%) | 0 (0%) | 2 (1.2%) |

## Part V - Congenital, familial and genetic disorders

| Congenital, familial and genetic disorders | ALEMTUZUMAB (n=8) | GLATIRAMER (n=23) | NATALIZUMAB (n=103) | OCRELIZUMAB (n=5) | Overall (n=139) |
|---|---|---|---|---|---|
| Accessory spleen | 0 (0%) | 0 (0%) | 1 (1.0%) | 0 (0%) | 1 (0.7%) |
| Adactyly | 0 (0%) | 1 (4.3%) | 0 (0%) | 0 (0%) | 1 (0.7%) |
| Amniotic band syndrome | 0 (0%) | 1 (4.3%) | 1 (1.0%) | 0 (0%) | 2 (1.4%) |
| Anencephaly | 0 (0%) | 0 (0%) | 2 (1.9%) | 0 (0%) | 2 (1.4%) |
| Ankyloglossia congenital | 0 (0%) | 0 (0%) | 1 (1.0%) | 0 (0%) | 1 (0.7%) |
| Anomaly of external ear congenital | 1 (12.5%) | 0 (0%) | 0 (0%) | 0 (0%) | 1 (0.7%) |
| Aorta hypoplasia | 0 (0%) | 0 (0%) | 1 (1.0%) | 0 (0%) | 1 (0.7%) |
| Atrial septal defect | 1 (12.5%) | 2 (8.7%) | 3 (2.9%) | 0 (0%) | 6 (4.3%) |
| Atrioventricular septal defect | 0 (0%) | 1 (4.3%) | 0 (0%) | 0 (0%) | 1 (0.7%) |
| Autosomal chromosome anomaly | 0 (0%) | 0 (0%) | 2 (1.9%) | 0 (0%) | 2 (1.4%) |
| Benign congenital hypotonia | 0 (0%) | 0 (0%) | 1 (1.0%) | 0 (0%) | 1 (0.7%) |
| Brachydactyly | 0 (0%) | 0 (0%) | 1 (1.0%) | 0 (0%) | 1 (0.7%) |
| Brain malformation | 0 (0%) | 0 (0%) | 1 (1.0%) | 0 (0%) | 1 (0.7%) |
| Cardiac malposition | 0 (0%) | 0 (0%) | 1 (1.0%) | 0 (0%) | 1 (0.7%) |
| Cardiac septal defect | 0 (0%) | 0 (0%) | 1 (1.0%) | 0 (0%) | 1 (0.7%) |
| Cerebellar hypoplasia | 0 (0%) | 0 (0%) | 1 (1.0%) | 0 (0%) | 1 (0.7%) |
| Cleft lip and palate | 0 (0%) | 2 (8.7%) | 0 (0%) | 0 (0%) | 2 (1.4%) |

| | | | | | |
|---|---|---|---|---|---|
| Cleft palate | 0 (0%) | 0 (0%) | 1 (1.0%) | 0 (0%) | 1 (0.7%) |
| Congenital anaemia | 0 (0%) | 0 (0%) | 1 (1.0%) | 0 (0%) | 1 (0.7%) |
| Congenital anomaly | 0 (0%) | 0 (0%) | 14 (13.6%) | 0 (0%) | 14 (10.1%) |
| Congenital anomaly of inner ear | 0 (0%) | 1 (4.3%) | 0 (0%) | 0 (0%) | 1 (0.7%) |
| Congenital aortic anomaly | 0 (0%) | 1 (4.3%) | 0 (0%) | 0 (0%) | 1 (0.7%) |
| Congenital cardiovascular anomaly | 0 (0%) | 0 (0%) | 1 (1.0%) | 0 (0%) | 1 (0.7%) |
| Congenital central nervous system anomaly | 0 (0%) | 0 (0%) | 2 (1.9%) | 0 (0%) | 2 (1.4%) |
| Congenital cerebral cyst | 0 (0%) | 0 (0%) | 1 (1.0%) | 0 (0%) | 1 (0.7%) |
| Congenital cystic kidney disease | 0 (0%) | 0 (0%) | 1 (1.0%) | 0 (0%) | 1 (0.7%) |
| Congenital cytomegalovirus infection | 1 (12.5%) | 0 (0%) | 0 (0%) | 0 (0%) | 1 (0.7%) |
| Congenital diaphragmatic hernia | 0 (0%) | 0 (0%) | 1 (1.0%) | 0 (0%) | 1 (0.7%) |
| Congenital foot malformation | 0 (0%) | 1 (4.3%) | 0 (0%) | 0 (0%) | 1 (0.7%) |
| Congenital gastric anomaly | 0 (0%) | 0 (0%) | 1 (1.0%) | 0 (0%) | 1 (0.7%) |
| Congenital hearing disorder | 0 (0%) | 1 (4.3%) | 0 (0%) | 0 (0%) | 1 (0.7%) |
| Congenital heart valve incompetence | 0 (0%) | 0 (0%) | 0 (0%) | 1 (20.0%) | 1 (0.7%) |
| Congenital hydrocephalus | 0 (0%) | 0 (0%) | 1 (1.0%) | 0 (0%) | 1 (0.7%) |
| Congenital hyperthyroidism | 1 (12.5%) | 0 (0%) | 0 (0%) | 0 (0%) | 1 (0.7%) |
| Congenital hypothyroidism | 1 (12.5%) | 0 (0%) | 0 (0%) | 0 (0%) | 1 (0.7%) |
| Congenital megacolon | 0 (0%) | 0 (0%) | 1 (1.0%) | 0 (0%) | 1 (0.7%) |
| Congenital naevus | 0 (0%) | 0 (0%) | 1 (1.0%) | 0 (0%) | 1 (0.7%) |
| Congenital pulmonary artery anomaly | 0 (0%) | 0 (0%) | 1 (1.0%) | 0 (0%) | 1 (0.7%) |
| Congenital pyelocaliectasis | 0 (0%) | 0 (0%) | 1 (1.0%) | 0 (0%) | 1 (0.7%) |
| Congenital renal disorder | 0 (0%) | 0 (0%) | 1 (1.0%) | 1 (20.0%) | 2 (1.4%) |
| Congenital skin disorder | 0 (0%) | 0 (0%) | 1 (1.0%) | 0 (0%) | 1 (0.7%) |
| Congenital thrombocytopenia | 0 (0%) | 0 (0%) | 2 (1.9%) | 0 (0%) | 2 (1.4%) |
| Congenital tricuspid valve atresia | 0 (0%) | 0 (0%) | 1 (1.0%) | 0 (0%) | 1 (0.7%) |
| Cryptorchism | 0 (0%) | 1 (4.3%) | 0 (0%) | 0 (0%) | 1 (0.7%) |
| Cytogenetic abnormality | 0 (0%) | 0 (0%) | 1 (1.0%) | 0 (0%) | 1 (0.7%) |
| Deafness congenital | 0 (0%) | 1 (4.3%) | 0 (0%) | 0 (0%) | 1 (0.7%) |

| | | | | | |
|---|---|---|---|---|---|
| Double outlet right ventricle | 0 (0%) | 0 (0%) | 1 (1.0%) | 0 (0%) | 1 (0.7%) |
| Exomphalos | 0 (0%) | 0 (0%) | 1 (1.0%) | 0 (0%) | 1 (0.7%) |
| Fallot's tetralogy | 0 (0%) | 0 (0%) | 1 (1.0%) | 0 (0%) | 1 (0.7%) |
| Gastroschisis | 0 (0%) | 0 (0%) | 1 (1.0%) | 0 (0%) | 1 (0.7%) |
| Gnathoschisis | 0 (0%) | 1 (4.3%) | 0 (0%) | 0 (0%) | 1 (0.7%) |
| Heart disease congenital | 0 (0%) | 0 (0%) | 3 (2.9%) | 0 (0%) | 3 (2.2%) |
| Heterotaxia | 0 (0%) | 0 (0%) | 2 (1.9%) | 0 (0%) | 2 (1.4%) |
| Hypospadias | 0 (0%) | 1 (4.3%) | 1 (1.0%) | 0 (0%) | 2 (1.4%) |
| Kidney malformation | 0 (0%) | 0 (0%) | 1 (1.0%) | 0 (0%) | 1 (0.7%) |
| Kinematic imbalances due to suboccipital strain | 0 (0%) | 1 (4.3%) | 0 (0%) | 0 (0%) | 1 (0.7%) |
| Macrocephaly | 0 (0%) | 0 (0%) | 1 (1.0%) | 0 (0%) | 1 (0.7%) |
| Meningocele | 0 (0%) | 0 (0%) | 1 (1.0%) | 0 (0%) | 1 (0.7%) |
| Microcephaly | 1 (12.5%) | 0 (0%) | 0 (0%) | 0 (0%) | 1 (0.7%) |
| Neuronal migration disorder | 0 (0%) | 0 (0%) | 1 (1.0%) | 0 (0%) | 1 (0.7%) |
| Otospondylomegaepiphyseal dysplasia | 0 (0%) | 1 (4.3%) | 1 (1.0%) | 0 (0%) | 2 (1.4%) |
| Patent ductus arteriosus | 0 (0%) | 1 (4.3%) | 2 (1.9%) | 0 (0%) | 3 (2.2%) |
| Phenylketonuria | 0 (0%) | 0 (0%) | 1 (1.0%) | 0 (0%) | 1 (0.7%) |
| Polydactyly | 0 (0%) | 2 (8.7%) | 0 (0%) | 1 (20.0%) | 3 (2.2%) |
| Porencephaly | 0 (0%) | 0 (0%) | 1 (1.0%) | 0 (0%) | 1 (0.7%) |
| Pulmonary artery atresia | 0 (0%) | 0 (0%) | 1 (1.0%) | 0 (0%) | 1 (0.7%) |
| Pulmonary sequestration | 0 (0%) | 0 (0%) | 1 (1.0%) | 0 (0%) | 1 (0.7%) |
| Pyloric stenosis | 2 (25.0%) | 0 (0%) | 0 (0%) | 0 (0%) | 2 (1.4%) |
| Renal aplasia | 0 (0%) | 0 (0%) | 1 (1.0%) | 0 (0%) | 1 (0.7%) |
| Respiratory tract malformation | 0 (0%) | 0 (0%) | 1 (1.0%) | 0 (0%) | 1 (0.7%) |
| Right aortic arch | 0 (0%) | 0 (0%) | 1 (1.0%) | 0 (0%) | 1 (0.7%) |
| Single umbilical artery | 0 (0%) | 0 (0%) | 1 (1.0%) | 0 (0%) | 1 (0.7%) |
| Skin hypoplasia | 0 (0%) | 0 (0%) | 1 (1.0%) | 0 (0%) | 1 (0.7%) |
| Spina bifida | 0 (0%) | 1 (4.3%) | 0 (0%) | 0 (0%) | 1 (0.7%) |
| Syndactyly | 0 (0%) | 0 (0%) | 1 (1.0%) | 0 (0%) | 1 (0.7%) |

| | | | | | |
|---|---|---|---|---|---|
| Talipes | 0 (0%) | 0 (0%) | 4 (3.9%) | 0 (0%) | 4 (2.9%) |
| Thalassaemia alpha | 0 (0%) | 0 (0%) | 1 (1.0%) | 0 (0%) | 1 (0.7%) |
| Thyroglossal cyst | 0 (0%) | 0 (0%) | 1 (1.0%) | 0 (0%) | 1 (0.7%) |
| Transposition of the great vessels | 0 (0%) | 1 (4.3%) | 0 (0%) | 0 (0%) | 1 (0.7%) |
| Trisomy 13 | 0 (0%) | 0 (0%) | 2 (1.9%) | 0 (0%) | 2 (1.4%) |
| Trisomy 18 | 0 (0%) | 0 (0%) | 3 (2.9%) | 0 (0%) | 3 (2.2%) |
| Trisomy 21 | 0 (0%) | 0 (0%) | 6 (5.8%) | 1 (20.0%) | 7 (5.0%) |
| Trisomy 9 | 0 (0%) | 0 (0%) | 1 (1.0%) | 0 (0%) | 1 (0.7%) |
| Turner's syndrome | 0 (0%) | 0 (0%) | 2 (1.9%) | 0 (0%) | 2 (1.4%) |
| Urethral valves | 0 (0%) | 0 (0%) | 0 (0%) | 1 (20.0%) | 1 (0.7%) |
| Ventricular hypoplasia | 0 (0%) | 0 (0%) | 1 (1.0%) | 0 (0%) | 1 (0.7%) |
| Ventricular septal defect | 0 (0%) | 1 (4.3%) | 4 (3.9%) | 0 (0%) | 5 (3.6%) |

## Part VI - Neonatal other ADRs

| Neonatal other ADR | ALEMTUZUMAB (N=18) | GLATIRAMER (N=3) | NATALIZUMAB (N=56) | OCRELIZUMAB (N=12) | Overall (N=89) |
|---|---|---|---|---|---|
| Adenovirus infection | 1 (5.6%) | 0 (0%) | 0 (0%) | 0 (0%) | 1 (1.1%) |
| Agitation | 1 (5.6%) | 0 (0%) | 0 (0%) | 0 (0%) | 1 (1.1%) |
| Anaemia | 0 (0%) | 0 (0%) | 4 (7.1%) | 0 (0%) | 4 (4.4%) |
| Anti-thyroid antibody positive | 2 (11.1%) | 0 (0%) | 0 (0%) | 0 (0%) | 2 (2.2%) |
| Areflexia | 1 (5.6%) | 0 (0%) | 0 (0%) | 0 (0%) | 1 (1.1%) |
| Arthropathy | 0 (0%) | 0 (0%) | 1 (1.8%) | 0 (0%) | 1 (1.1%) |
| Autism spectrum disorder | 0 (0%) | 0 (0%) | 2 (3.6%) | 0 (0%) | 2 (2.2%) |

| | | | | | |
|---|---|---|---|---|---|
| Blood bilirubin decreased | 0 (0%) | 0 (0%) | 1 (1.8%) | 0 (0%) | 1 (1.1%) |
| Blood glucose decreased | 0 (0%) | 0 (0%) | 0 (0%) | 1 (8.3%) | 1 (1.1%) |
| Blood test abnormal | 0 (0%) | 0 (0%) | 1 (1.8%) | 0 (0%) | 1 (1.1%) |
| Cardiac failure | 0 (0%) | 0 (0%) | 1 (1.8%) | 0 (0%) | 1 (1.1%) |
| Cardiac murmur | 0 (0%) | 0 (0%) | 2 (3.6%) | 0 (0%) | 2 (2.2%) |
| Cerebrovascular accident | 0 (0%) | 0 (0%) | 0 (0%) | 1 (8.3%) | 1 (1.1%) |
| Congenital musculoskeletal disorder | 0 (0%) | 0 (0%) | 0 (0%) | 1 (8.3%) | 1 (1.1%) |
| Constipation | 0 (0%) | 0 (0%) | 1 (1.8%) | 0 (0%) | 1 (1.1%) |
| Cystic lung disease | 0 (0%) | 0 (0%) | 1 (1.8%) | 0 (0%) | 1 (1.1%) |
| Cytomegalovirus infection reactivation | 1 (5.6%) | 0 (0%) | 0 (0%) | 0 (0%) | 1 (1.1%) |
| Enterococcus test positive | 0 (0%) | 0 (0%) | 1 (1.8%) | 0 (0%) | 1 (1.1%) |
| Escherichia sepsis | 0 (0%) | 0 (0%) | 1 (1.8%) | 0 (0%) | 1 (1.1%) |
| Eyelid disorder | 0 (0%) | 0 (0%) | 0 (0%) | 1 (8.3%) | 1 (1.1%) |
| Feeding disorder | 0 (0%) | 0 (0%) | 1 (1.8%) | 0 (0%) | 1 (1.1%) |
| Fluid intake reduced | 0 (0%) | 0 (0%) | 0 (0%) | 0 (0%) | 1 (1.1%) |
| Foot deformity | 0 (0%) | 0 (0%) | 1 (1.8%) | 0 (0%) | 1 (1.1%) |
| Granulocyte count decreased | 0 (0%) | 0 (0%) | 1 (1.8%) | 0 (0%) | 1 (1.1%) |
| Haemangioma | 0 (0%) | 0 (0%) | 2 (3.6%) | 0 (0%) | 2 (2.2%) |
| Haematochezia | 0 (0%) | 0 (0%) | 1 (1.8%) | 0 (0%) | 1 (1.1%) |
| Haemoglobin abnormal | 0 (0%) | 0 (0%) | 1 (1.8%) | 0 (0%) | 1 (1.1%) |
| Haemoglobin decreased | 0 (0%) | 0 (0%) | 1 (1.8%) | 0 (0%) | 1 (1.1%) |
| Heart rate decreased | 0 (0%) | 0 (0%) | 0 (0%) | 1 (8.3%) | 1 (1.1%) |
| Hypertension | 1 (5.6%) | 0 (0%) | 0 (0%) | 0 (0%) | 1 (1.1%) |
| Hyponatraemia | 1 (5.6%) | 0 (0%) | 0 (0%) | 0 (0%) | 1 (1.1%) |
| Hypophagia | 0 (0%) | 0 (0%) | 1 (1.8%) | 0 (0%) | 1 (1.1%) |
| Hypotonia | 0 (0%) | 1 (33.3%) | 0 (0%) | 0 (0%) | 1 (1.1%) |
| Ileal perforation | 0 (0%) | 0 (0%) | 1 (1.8%) | 0 (0%) | 1 (1.1%) |
| Infection | 0 (0%) | 0 (0%) | 1 (1.8%) | 0 (0%) | 1 (1.1%) |
| Inguinal hernia | 1 (5.6%) | 0 (0%) | 0 (0%) | 0 (0%) | 1 (1.1%) |

| | | | | | |
|---|---|---|---|---|---|
| Jaundice | 0 (0%) | 0 (0%) | 1 (1.8%) | 0 (0%) | 1 (1.1%) |
| Leukocytosis | 0 (0%) | 0 (0%) | 1 (1.8%) | 0 (0%) | 1 (1.1%) |
| Long QT syndrome | 0 (0%) | 0 (0%) | 0 (0%) | 1 (8.3%) | 1 (1.1%) |
| Loss of consciousness | 0 (0%) | 1 (33.3%) | 0 (0%) | 0 (0%) | 1 (1.1%) |
| Meningitis bacterial | 0 (0%) | 0 (0%) | 1 (1.8%) | 0 (0%) | 1 (1.1%) |
| Mitral valve incompetence | 1 (5.6%) | 0 (0%) | 0 (0%) | 0 (0%) | 1 (1.1%) |
| Multiple organ dysfunction syndrome | 0 (0%) | 1 (33.3%) | 0 (0%) | 0 (0%) | 1 (1.1%) |
| Muscle tone disorder | 1 (5.6%) | 0 (0%) | 0 (0%) | 0 (0%) | 1 (1.1%) |
| Oxygen saturation decreased | 0 (0%) | 0 (0%) | 1 (1.8%) | 0 (0%) | 1 (1.1%) |
| Pancreatitis | 1 (5.6%) | 0 (0%) | 0 (0%) | 0 (0%) | 1 (1.1%) |
| Petechiae | 0 (0%) | 0 (0%) | 1 (1.8%) | 0 (0%) | 1 (1.1%) |
| Platelet count decreased | 0 (0%) | 0 (0%) | 3 (5.4%) | 0 (0%) | 3 (3.3%) |
| Pneumonia | 0 (0%) | 0 (0%) | 0 (0%) | 1 (8.3%) | 1 (1.1%) |
| Pneumothorax | 0 (0%) | 0 (0%) | 0 (0%) | 1 (8.3%) | 1 (1.1%) |
| Posterior reversible encephalopathy syndrome | 1 (5.6%) | 0 (0%) | 0 (0%) | 0 (0%) | 1 (1.1%) |
| Pulmonary arterial hypertension | 0 (0%) | 0 (0%) | 1 (1.8%) | 0 (0%) | 1 (1.1%) |
| Pulmonary sepsis | 0 (0%) | 0 (0%) | 0 (0%) | 1 (8.3%) | 1 (1.1%) |
| Pulmonary valve stenosis | 0 (0%) | 0 (0%) | 2 (3.6%) | 0 (0%) | 2 (2.2%) |
| Pulmonary vein stenosis | 0 (0%) | 0 (0%) | 1 (1.8%) | 0 (0%) | 1 (1.1%) |
| Reduced facial expression | 1 (5.6%) | 0 (0%) | 0 (0%) | 0 (0%) | 1 (1.1%) |
| Respiratory disorder | 1 (5.6%) | 0 (0%) | 0 (0%) | 0 (0%) | 1 (1.1%) |
| Respiratory distress | 0 (0%) | 0 (0%) | 0 (0%) | 1 (8.3%) | 1 (1.1%) |
| Respiratory failure | 0 (0%) | 0 (0%) | 1 (1.8%) | 0 (0%) | 1 (1.1%) |
| Respiratory syncytial virus bronchiolitis | 0 (0%) | 0 (0%) | 0 (0%) | 1 (8.3%) | 1 (1.1%) |
| Retinal haemorrhage | 0 (0%) | 0 (0%) | 1 (1.8%) | 0 (0%) | 1 (1.1%) |
| Seizure | 0 (0%) | 0 (0%) | 1 (1.8%) | 0 (0%) | 1 (1.1%) |
| Sepsis | 0 (0%) | 0 (0%) | 0 (0%) | 1 (8.3%) | 1 (1.1%) |
| Serum ferritin increased | 0 (0%) | 0 (0%) | 1 (1.8%) | 0 (0%) | 1 (1.1%) |
| Short stature | 1 (5.6%) | 0 (0%) | 0 (0%) | 0 (0%) | 1 (1.1%) |

| | | | | | |
|---|---|---|---|---|---|
| Staphylococcus test positive | 0 (0%) | 0 (0%) | 1 (1.8%) | 0 (0%) | 1 (1.1%) |
| Tachycardia | 1 (5.6%) | 0 (0%) | 0 (0%) | 0 (0%) | 1 (1.1%) |
| Thrombocytopenia | 0 (0%) | 0 (0%) | 9 (16.1%) | 0 (0%) | 9 (10.0%) |
| Weight decreased | 0 (0%) | 0 (0%) | 1 (1.8%) | 0 (0%) | 1 (1.1%) |
| Weight gain poor | 0 (0%) | 0 (0%) | 1 (1.8%) | 0 (0%) | 1 (1.1%) |
| White blood cell disorder | 0 (0%) | 0 (0%) | 1 (1.8%) | 0 (0%) | 1 (1.1%) |
| Wound infection | 1 (5.6%) | 0 (0%) | 0 (0%) | 0 (0%) | 1 (1.1%) |

## Part VII – Foetal disorders

| Foetal disorders | ALEMTUZUMAB (N=4) | GLATIRAMER (N=12) | NATALIZUMAB (N=36) | OCRELIZUMAB (N=1) | Overall (N=53) |
|---|---|---|---|---|---|
| Bradycardia foetal | 0 (0%) | 0 (0%) | 2 (5.6%) | 0 (0%) | 2 (3.8%) |
| Foetal anaemia | 0 (0%) | 0 (0%) | 3 (8.3%) | 0 (0%) | 3 (5.7%) |
| Foetal disorder | 1 (25.0%) | 0 (0%) | 2 (5.6%) | 0 (0%) | 3 (5.7%) |
| Foetal distress syndrome | 0 (0%) | 0 (0%) | 4 (11.1%) | 0 (0%) | 4 (7.5%) |
| Foetal growth restriction | 3 (75.0%) | 5 (41.7%) | 10 (27.8%) | 1 (100%) | 19 (35.8%) |
| Foetal heart rate abnormal | 0 (0%) | 2 (16.7%) | 5 (13.9%) | 0 (0%) | 7 (13.2%) |
| Foetal heart rate deceleration abnormality | 0 (0%) | 0 (0%) | 2 (5.6%) | 0 (0%) | 2 (3.8%) |
| Foetal heart rate decreased | 0 (0%) | 2 (16.7%) | 1 (2.8%) | 0 (0%) | 3 (5.7%) |
| Foetal malformation | 0 (0%) | 2 (16.7%) | 5 (13.9%) | 0 (0%) | 7 (13.2%) |
| Hydrops foetalis | 0 (0%) | 0 (0%) | 2 (5.6%) | 0 (0%) | 2 (3.8%) |
| Meconium in amniotic fluid | 0 (0%) | 1 (8.3%) | 0 (0%) | 0 (0%) | 1 (1.9%) |

**Part VIII - Infant other ADRs**

| Infant other ADR | ALEMTUZUMAB (N=5) | GLATIRAMER (N=9) | NATALIZUMAB (N=20) | OCRELIZUMAB (N=10) | Overall (N=44) |
|---|---|---|---|---|---|
| Anaemia | 0 (0%) | 0 (0%) | 3 (15.0%) | 0 (0%) | 3 (6.8%) |
| Autism spectrum disorder | 0 (0%) | 0 (0%) | 3 (15.0%) | 0 (0%) | 3 (6.8%) |
| Autoimmune neutropenia | 0 (0%) | 0 (0%) | 1 (5.0%) | 0 (0%) | 1 (2.3%) |
| Blood thyroid stimulating hormone decreased | 2 (40.0%) | 0 (0%) | 0 (0%) | 0 (0%) | 2 (4.5%) |
| Bronchitis | 0 (0%) | 1 (11.1%) | 0 (0%) | 0 (0%) | 1 (2.3%) |
| Cough | 0 (0%) | 0 (0%) | 1 (5.0%) | 0 (0%) | 1 (2.3%) |
| COVID-19 | 0 (0%) | 2 (22.2%) | 0 (0%) | 1 (10.0%) | 3 (6.8%) |
| Deafness unilateral | 0 (0%) | 0 (0%) | 1 (5.0%) | 0 (0%) | 1 (2.3%) |
| Enterococcal infection | 0 (0%) | 0 (0%) | 0 (0%) | 1 (10.0%) | 1 (2.3%) |
| Gastroenteritis | 0 (0%) | 0 (0%) | 1 (5.0%) | 1 (10.0%) | 2 (4.5%) |
| Haemangioma of skin | 0 (0%) | 0 (0%) | 1 (5.0%) | 0 (0%) | 1 (2.3%) |
| Heart sounds abnormal | 0 (0%) | 1 (11.1%) | 0 (0%) | 0 (0%) | 1 (2.3%) |
| Hypersensitivity | 0 (0%) | 0 (0%) | 1 (5.0%) | 0 (0%) | 1 (2.3%) |
| Infection | 0 (0%) | 1 (11.1%) | 0 (0%) | 0 (0%) | 1 (2.3%) |
| Influenza | 0 (0%) | 0 (0%) | 0 (0%) | 1 (10.0%) | 1 (2.3%) |
| Lactose intolerance | 0 (0%) | 0 (0%) | 1 (5.0%) | 0 (0%) | 1 (2.3%) |
| Malaise | 0 (0%) | 0 (0%) | 0 (0%) | 1 (10.0%) | 1 (2.3%) |
| Myoclonic epilepsy | 0 (0%) | 0 (0%) | 1 (5.0%) | 0 (0%) | 1 (2.3%) |
| Normal newborn | 0 (0%) | 0 (0%) | 1 (5.0%) | 0 (0%) | 1 (2.3%) |
| Pain | 0 (0%) | 0 (0%) | 0 (0%) | 1 (10.0%) | 1 (2.3%) |
| Pyelonephritis | 0 (0%) | 0 (0%) | 1 (5.0%) | 0 (0%) | 1 (2.3%) |
| Pyrexia | 0 (0%) | 1 (11.1%) | 0 (0%) | 1 (10.0%) | 2 (4.5%) |
| Respiratory syncytial virus infection | 0 (0%) | 0 (0%) | 0 (0%) | 1 (10.0%) | 1 (2.3%) |
| Sensitisation | 0 (0%) | 0 (0%) | 1 (5.0%) | 0 (0%) | 1 (2.3%) |
| Speech disorder developmental | 0 (0%) | 0 (0%) | 1 (5.0%) | 0 (0%) | 1 (2.3%) |

| | | | | | |
|---|---|---|---|---|---|
| Staphylococcal infection | 0 (0%) | 0 (0%) | 0 (0%) | 1 (10.0%) | 1 (2.3%) |
| Stridor | 0 (0%) | 1 (11.1%) | 0 (0%) | 0 (0%) | 1 (2.3%) |
| Thrombocytopenia | 0 (0%) | 0 (0%) | 1 (5.0%) | 0 (0%) | 1 (2.3%) |
| Thyroid disorder | 1 (20.0%) | 0 (0%) | 0 (0%) | 0 (0%) | 1 (2.3%) |
| Thyroid function test abnormal | 2 (40.0%) | 0 (0%) | 0 (0%) | 0 (0%) | 2 (4.5%) |
| Underweight | 0 (0%) | 0 (0%) | 1 (5.0%) | 0 (0%) | 1 (2.3%) |
| Viral infection | 0 (0%) | 1 (11.1%) | 0 (0%) | 0 (0%) | 1 (2.3%) |
| Vomiting | 0 (0%) | 0 (0%) | 0 (0%) | 1 (10.0%) | 1 (2.3%) |
| Weight gain poor | 0 (0%) | 1 (11.1%) | 0 (0%) | 0 (0%) | 1 (2.3%) |

**Part IX - Foetal other ADRs**

| Foetal disorders | ALEMTUZUMAB (N=6) | GLATIRAMER (N=0) | NATALIZUMAB (N=0) | OCRELIZUMAB (N=4) | Overall (N=10) |
|---|---|---|---|---|---|
| Bladder dilatation | 0 (0%) | 0 (0%) | 0 (0%) | 1 (25.0%) | 1 (10.0%) |
| Cerebral calcification | 1 (16.7%) | 0 (0%) | 0 (0%) | 0 (0%) | 1 (10.0%) |
| Cerebral cyst | 1 (16.7%) | 0 (0%) | 0 (0%) | 0 (0%) | 1 (10.0%) |
| Cerebral ventricle dilatation | 1 (16.7%) | 0 (0%) | 0 (0%) | 0 (0%) | 1 (10.0%) |
| Congenital musculoskeletal disorder of skull | 0 (0%) | 0 (0%) | 0 (0%) | 1 (25.0%) | 1 (10.0%) |
| Deafness unilateral | 1 (16.7%) | 0 (0%) | 0 (0%) | 0 (0%) | 1 (10.0%) |
| Hydronephrosis | 0 (0%) | 0 (0%) | 0 (0%) | 1 (25.0%) | 1 (10.0%) |
| Hyperthyroidism | 1 (16.7%) | 0 (0%) | 0 (0%) | 0 (0%) | 1 (10.0%) |
| Hypertonia | 1 (16.7%) | 0 (0%) | 0 (0%) | 0 (0%) | 1 (10.0%) |
| Weight increased | 0 (0%) | 0 (0%) | 0 (0%) | 1 (25.0%) | 1 (10.0%) |

**Part X - PTs not indicating ADR**

| PT not indicating ADR | ALEMTUZUMAB (N=89) | GLATIRAMER (N=292) | NATALIZUMAB (N=844) | OCRELIZUMAB (N=101) | Overall (N=1,326) |
|---|---|---|---|---|---|
| Adverse drug reaction | 1 (1.1%) | 0 (0%) | 0 (0%) | 0 (0%) | 1 (0.1%) |
| Adverse event | 1 (1.1%) | 0 (0%) | 0 (0%) | 0 (0%) | 1 (0.1%) |
| Drug exposure before pregnancy | 20 (22.5%) | 1 (0.3%) | 0 (0%) | 0 (0%) | 21 (1.6%) |
| Drug monitoring procedure not performed | 1 (1.1%) | 0 (0%) | 0 (0%) | 0 (0%) | 1 (0.1%) |
| Exposure during pregnancy | 28 (31.5%) | 9 (3.1%) | 3 (0.4%) | 2 (2.0%) | 42 (3.2%) |
| Exposure via breast milk | 3 (3.4%) | 6 (2.1%) | 21 (2.5%) | 2 (2.0%) | 32 (2.4%) |
| Foetal exposure during pregnancy | 7 (7.9%) | 37 (12.7%) | 179 (21.2%) | 22 (21.8%) | 245 (18.5%) |
| Foetal exposure timing unspecified | 4 (4.5%) | 0 (0%) | 2 (0.2%) | 0 (0%) | 6 (0.5%) |
| Incorrect drug administration rate | 1 (1.1%) | 0 (0%) | 0 (0%) | 0 (0%) | 1 (0.1%) |
| Intentional product use issue | 1 (1.1%) | 0 (0%) | 2 (0.2%) | 1 (1.0%) | 4 (0.3%) |
| Maternal exposure before pregnancy | 2 (2.2%) | 3 (1.0%) | 0 (0%) | 20 (19.8%) | 25 (1.9%) |
| Maternal exposure during pregnancy | 4 (4.5%) | 134 (45.9%) | 588 (69.7%) | 35 (34.7%) | 761 (57.4%) |
| Normal newborn | 1 (1.1%) | 39 (13.4%) | 0 (0%) | 0 (0%) | 40 (3.0%) |
| Poor venous access | 1 (1.1%) | 0 (0%) | 0 (0%) | 0 (0%) | 1 (0.1%) |
| Pregnancy | 14 (15.7%) | 11 (3.8%) | 2 (0.2%) | 4 (4.0%) | 31 (2.3%) |
| Accidental exposure to product | 0 (0%) | 1 (0.3%) | 0 (0%) | 0 (0%) | 1 (0.1%) |
| Foetal monitoring | 0 (0%) | 1 (0.3%) | 0 (0%) | 0 (0%) | 1 (0.1%) |
| Illness | 0 (0%) | 1 (0.3%) | 0 (0%) | 0 (0%) | 1 (0.1%) |
| Inappropriate schedule of product administration | 0 (0%) | 1 (0.3%) | 1 (0.1%) | 0 (0%) | 2 (0.2%) |
| Infant | 0 (0%) | 1 (0.3%) | 0 (0%) | 0 (0%) | 1 (0.1%) |
| Intentional product misuse | 0 (0%) | 1 (0.3%) | 0 (0%) | 0 (0%) | 1 (0.1%) |
| Live birth | 0 (0%) | 19 (6.5%) | 0 (0%) | 0 (0%) | 19 (1.4%) |
| Maternal exposure during breast feeding | 0 (0%) | 20 (6.8%) | 2 (0.2%) | 1 (1.0%) | 23 (1.7%) |
| Pregnancy test positive | 0 (0%) | 2 (0.7%) | 0 (0%) | 0 (0%) | 2 (0.2%) |

| | | | | | |
|---|---|---|---|---|---|
| Product use in unapproved indication | 0 (0%) | 1 (0.3%) | 1 (0.1%) | 0 (0%) | 2 (0.2%) |
| Therapy interrupted | 0 (0%) | 1 (0.3%) | 0 (0%) | 0 (0%) | 1 (0.1%) |
| Wrong technique in product usage process | 0 (0%) | 3 (1.0%) | 0 (0%) | 0 (0%) | 3 (0.2%) |
| Off label use | 0 (0%) | 0 (0%) | 3 (0.4%) | 4 (4.0%) | 7 (0.5%) |
| Prescribed underdose | 0 (0%) | 0 (0%) | 38 (4.5%) | 0 (0%) | 38 (2.9%) |
| Product dose omission issue | 0 (0%) | 0 (0%) | 1 (0.1%) | 0 (0%) | 1 (0.1%) |
| Road traffic accident | 0 (0%) | 0 (0%) | 1 (0.1%) | 0 (0%) | 1 (0.1%) |
| Drug interaction | 0 (0%) | 0 (0%) | 0 (0%) | 1 (1.0%) | 1 (0.1%) |
| Maternal exposure timing unspecified | 0 (0%) | 0 (0%) | 0 (0%) | 8 (7.9%) | 8 (0.6%) |
| Product storage error | 0 (0%) | 0 (0%) | 0 (0%) | 1 (1.0%) | 1 (0.1%) |
